# Obesity‐Related Coagulation Activation in Adolescents and Children: A Systematic Review and Meta‐Analysis

**DOI:** 10.1111/obr.70137

**Published:** 2026-04-26

**Authors:** Julia Buchold, Hans‐Jonas Meyer, Manuel Florian Struck, Shrey Kohli, Akash Mathew, Gyulten Mangova, Alexey Surov, Matthias Blüher, Berend Isermann, Silke Zimmermann

**Affiliations:** ^1^ Institute of Laboratory Medicine, Clinical Chemistry and Molecular Diagnostics University of Leipzig Leipzig Germany; ^2^ Department of Diagnostic and Interventional Radiology University of Leipzig Leipzig Germany; ^3^ Department of Anesthesiology and Intensive Care Medicine University of Leipzig Leipzig Germany; ^4^ Department of Radiology, Neuroradiology and Nuclear Medicine Johannes Wesling University Hospital, Ruhr University Bochum Germany; ^5^ Helmholtz Institute for Metabolic Obesity and Vascular Research (HI‐MAG) Helmholtz Zentrum München, University of Leipzig and University Hospital Leipzig Leipzig Germany; ^6^ Medical Department III—Endocrinology, Nephrology, and Rheumatology Leipzig University Medical Center Leipzig Germany; ^7^ German Center for Child and Adolescent Health (DZKJ), Leipzig/Dresden Partner Site Leipzig Germany; ^8^ LeiCeM—Leipzig Center of Metabolism Leipzig University Leipzig Germany

**Keywords:** coagulation activation, meta‐analysis, obesity, systematic review

## Abstract

Obesity is recognized as a pro‐thrombotic condition, yet the extent of coagulation activation across biomarkers remains unclear. This meta‐analysis evaluates the impact of obesity on parameters—D‐dimer, fibrinogen, plasminogen activator inhibitor‐1 (PAI‐1), von Willebrand factor (vWF), factor VIII (FVIII), and endogenous thrombin potential (ETP)—in children and adults.

**Methods:**

Sixty‐four studies comprising 59,503 individuals were analyzed. Plasma biomarker levels were compared between non‐obese and obese groups using standardized mean differences (SMDs), with subgroup analyses.

**Results:**

D‐dimer was significantly elevated in adults with obesity (SMD 1.36, 95% CI 0.47–2.25, *p* = 0.003) and children with obesity (SMD 0.77, 95% CI 0.19–1.36, *p* = 0.009), indicating increased fibrin turnover. Fibrinogen levels were markedly higher in both adults (SMD 1.17, 95% CI 0.14–2.20, *p* = 0.03) and children (SMD 1.43, 95% CI 0.93–1.92, *p* < 0.0001). PAI‐1 showed the most pronounced increase in adults (SMD 2.30, 95% CI 0.1.51–3.09, *p* < 0.0001) and children (SMD 3.54, 95% CI 1.65–5.43, *p* = 0.0002). FVIII levels were modestly elevated (SMD 0.52, 95% CI 0.10–0.94, *p* = 0.02), whereas vWF levels showed inconsistent changes. ETP was significantly higher in obesity, in children (SMD 1.06, 95% CI 0.24–1.88, *p* = 0.01) and adults (SMD 0.71, 95% CI 0.46–0.97, *p* < 0.0001). Gender‐stratified data indicated higher PAI‐1, fibrinogen, and ETP levels in females.

**Conclusion:**

Obesity is associated with increased coagulation activation, suggesting a pro‐thrombotic shift. These findings support the need for age‐ and gender‐specific research into obesity‐related hemostatic alterations.

## Graphical abstract

Obesity is widely recognized as a pro‐thrombotic condition, yet the specific biomarker profile reflecting coagulation activation remains incompletely defined. Obesity is associated with increased coagulation activation, particularly in children, suggesting an early pro‐thrombotic shift.

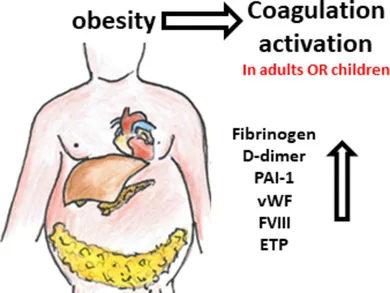

## 1 Introduction

The global rise in obesity represents a major public health concern due to its adverse effects on quality of life and life expectancy. Excess body weight is now recognized as one of the leading causes of death and disability both in the United States and worldwide, with the associated burden projected to escalate in the coming years [1]. Obesity is not only associated with traditional cardiovascular risk factors—such as hypertension, type 2 diabetes, and dyslipidemia—but also with a prothrombotic state driven by complex metabolic and inflammatory processes [2].

The accumulation of adipose tissue, particularly visceral fat, has emerged as a key contributor to the pathogenesis of thrombotic vascular diseases. Beyond mechanical and metabolic impacts, adipose tissue functions as an endocrine organ with secretory activity that influences vascular homeostasis and hemostasis. Dysregulation of this secretory profile can alter coagulation pathways and enhance thrombogenicity [2]. These effects are mediated in part by adipocyte‐derived cytokines and coagulation‐modifying proteins.

Obesity increases the levels of several procoagulant factors while concurrently suppressing fibrinolytic activity, thereby promoting a hypercoagulable state [3, 4]. Notably, sex‐specific differences in coagulation responses to obesity have been reported. For instance, the Hoorn Study demonstrated that obesity and abdominal fat were associated with increased thrombin generation in women, but not in men, suggesting sexually dimorphic effects of adiposity on coagulation [5, 6].

Among the key biomarkers of coagulation and fibrinolysis, D‐dimer reflects both fibrin formation and degradation, serving as a marker of thrombosis and fibrinolysis. Additionally, as inflammation is typically associated with increased thrombogenicity, D‐dimer as well as other coagulation factors are associated with inflammation. Elevated D‐dimer levels have been consistently observed in individuals with obesity, indicating ongoing coagulation activation and enhanced fibrin formation and dissolution [7].

An important regulator of fibrinolysis is plasminogen activator inhibitor‐1 (PAI‐1), the primary inhibitor of tissue‐type and urokinase‐type plasminogen activators (tPA and uPA). It is produced by multiple cell types including adipocytes, hepatocytes, platelets, vascular smooth muscle cells, fibroblasts, and immune cells. Under pathological conditions, tumor and inflammatory cells also contribute to PAI‐1 expression [8]. Elevated PAI‐1 levels in obesity may result from both genetic predisposition and environmental or physiological factors such as inflammation, hypoxia, age, BMI, and metabolic derangements. Increased PAI‐1 concentrations have been identified in individuals with obesity compared with controls, implicating it as a mechanistic link between adiposity and hypercoagulability [9].

An established marker of hypercoagulability is the endogenous thrombin potential (ETP), which quantifies the capacity of plasma to generate thrombin over time. It represents the area under the thrombin generation curve and is particularly sensitive to alterations in procoagulant and anticoagulant balance. Prüller et al. demonstrated an association between trunk fat obesity and increased ETP in asymptomatic adults [10]. Recent advancements in thrombin generation assays now allow detailed characterization of coagulation dynamics under varying conditions, facilitating the assessment of how external factors, including obesity, impact different components of the coagulation cascade [11, 12].

In addition to adults, the adverse hemostatic effects of obesity have also been observed in pediatric populations. Given that cardiovascular risk factors often originate in childhood, early manifestations of altered hemostasis may have long‐term implications. Visceral adiposity in children has been associated with elevated levels of fibrinogen, a key determinant of plasma viscosity and erythrocyte aggregation. Elevated fibrinogen may impair microcirculatory flow, damage endothelium, and predispose to thrombosis [13, 14]. Importantly, fibrinogen levels have been shown to decline in response to weight loss interventions such as caloric restriction [15]. The biological mechanisms linking central obesity to elevated fibrinogen likely involve hepatic synthesis stimulated by pro‐inflammatory cytokines—especially interleukin‐6, which is secreted in greater quantities by abdominal adipose tissue in individuals with obesity [16, 17]. Other mechanisms may include altered fibrinogen metabolism or clearance [18, 19].

Although the association of hypercoagulability with obesity has been frequently proposed, a comprehensive evaluation of the relationship between obesity and hemostatic activation is lacking. Hence, we conducted a systematic review and meta‐analysis investigating obesity‐associated alterations in six thrombosis‐related biomarkers: fibrinogen, D‐dimer, PAI‐1, von Willebrand factor (vWF), factor VIII (FVIII), and endogenous thrombin potential (ETP).

## 2 Methods

### 2.1 Search Strategy

Including data until February 2025, a comprehensive search of worldwide web databases, including PubMed and Web of Science, was conducted. The keywords utilized in the continuous analysis were “obesity” OR “adiposity” AND “fibrinogen” OR “thrombin” OR “D‐dimer” OR “plasminogen activator inhibitor 1” OR “endogenous thrombin potential” OR “Factor VIII” OR “von Willebrand Factor” OR “Factor VII” OR “Factor XIII” OR “Factor V" in addition to other pertinent keywords. The search was performed using filters for “full text” or “free full text.”

### 2.2 Inclusion and Exclusion Criteria

The inclusion criteria were (1) studies assessing the levels of D‐dimer, fibrinogen, PAI‐1, vWF, FVIII, ETP, FXIII, FVII, and FV in obesity in children or adults. We excluded (1) studies without a control group; (2) studies without standard deviation or cut‐offs or non‐convertible units; (3) non‐English articles; and (4) letters, commentaries, case reports, conference abstracts, meta‐analyses, and reviews.

### 2.3 Screening

Two reviewers (S.Z. and J.B.) independently reviewed titles and abstracts for relevant articles based on inclusion and exclusion criteria, after eliminating duplicates from the initial search. In addition, manuscripts referenced in identified publications were considered.

### 2.4 Data Extraction

Using a data extraction sheet, two independent reviewers (S.Z. and J.B.) extracted the following information from each study: (1) first author's name, publication year, and country of conduct; (2) the demographic characteristics of the cases (sample size, mean age, and gender distribution in each obese and control group).

### 2.5 Quality Assessment

The “Newcastle–Ottawa Quality Assessment Scale” (NOS) for observational studies was used for the quality assessment of included studies (https://www.ohri.ca/programs/clinical_epidemiology/oxford.asp). Two researchers (H.J.M. and J.B.) conducted the quality evaluation individually and mainly included the selection of cases, comparability of cohorts, and outcome assessment. A score of 0–9 was assigned to each study. A study with ≥ 6 was considered to be of high quality.

### 2.6 Statistical Analysis

The meta‐analysis was performed using RevMan 5.4 [20]. Heterogeneity was calculated by means of the inconsistency index *I* ^2^ [21, 22]. Random effect meta‐analysis was performed to calculate the standardized mean difference (SMD) and 95% confidence interval (CI). Whenever interquartile range was mentioned instead of mean, the following formula was applied: (Q3 − Q1)/1349 [23].

### 2.7 Publication Bias Assessment and Assay Variability

Funnel plots and Egger's regression test were used to assess publication bias when sufficient studies were available (≥ 10) [24]. Sensitivity analyses excluding studies with alternative or unclear methods were conducted to assess the stability of the results.

## 3 Results

### 3.1 Literature Search and Baseline Characteristics of Included Studies

**FIGURE 1:**
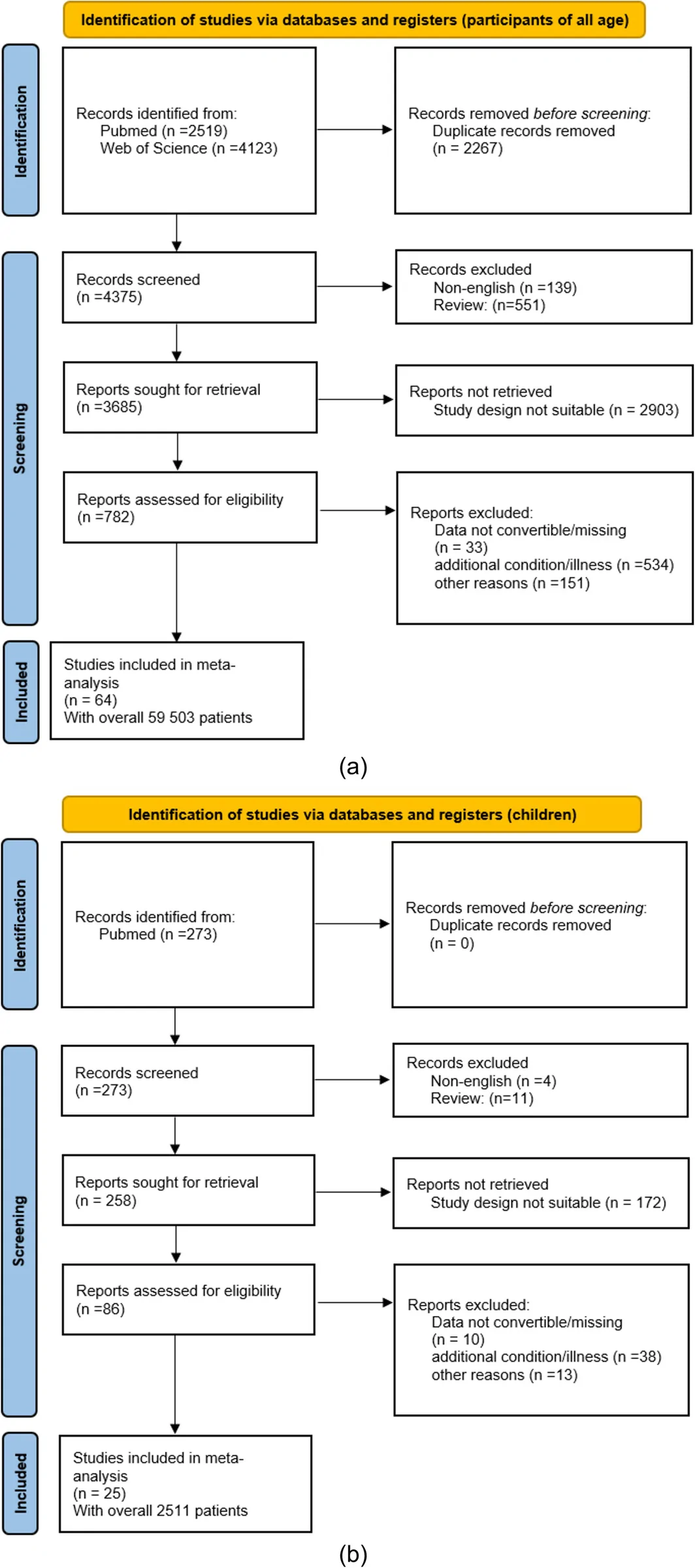
(a) Flow diagram summarizing the selection of eligible studies in adults based on the PRISMA guidelines. Sixty‐four studies with 59,503 participants (adults and children) were included in our meta‐analysis that measured levels of D‐dimer, fibrinogen, plasminogen activator inhibitor 1 (PAI‐1), von Willebrand factor (vWF), FVIII, or endogenous thrombin potential (ETP) in adults and children. (b) Flow diagram summarizing the selection of eligible studies in children based on the PRISMA guidelines. Twenty‐five studies with 2511 participants (only children) were included in our meta‐analysis that measured levels of D‐dimer, fibrinogen, plasminogen activator inhibitor 1 (PAI‐1), von Willebrand factor (vWF), FVIII, or endogenous thrombin potential (ETP).

We included 6642 records from PubMed (*n* = 2519, Figure 1) and Web of Science (*n* = 4123, Figure 1) based on the PRISMA guidelines [25]. After removing 2267 (Figure 1) duplicates, further records were excluded based on title and abstract screening. We identified studies based on the following criteria: studies related to outcome, English‐written articles, and studies including a suitable control group (Figure 1). We included studies with the continuous variable “fibrinogen,” “d‐dimer,” “ETP,” “FVIII,” “vWF,” or “PAI‐1” in obesity (random effect meta‐analysis, Figure 1). Studies included were conducted between 2002 and 2024 (Table 1). After thorough review, 64 studies were found to be suitable for the present analysis [7, 26, 27, 28, 29, 30, 31, 32, 33, 34, 35, 36, 37, 38, 39, 40, 41, 42, 43, 44, 45, 46, 47, 48, 49, 50, 51, 52, 53, 54, 55, 56, 57, 58, 59, 60, 61, 62, 63, 64, 65, 66, 67, 68, 69, 70, 71, 72, 73, 74, 75, 76, 77, 78, 79, 80, 81, 82, 83, 84, 85, 86].

**TABLE 1:** Overview of the included studies.

Authors	Country	Study design	Included patients, *n*	Mean age, years	BMI in the obesity group	Gender, female, *n* (%)
Firszt‐Adamczyk, 2016	Poland	Controlled before after study (only baseline data used)	60	40	> 40	70
Ceo, 2012	Brazil	Cross‐sectional	39	38	39	100
Michalska, 2013	Poland	Cross‐sectional	70	38	48.5	60
Kaye, 2012	Finland	Population‐based, longitudinal study, multicenter	28	25	30.5	42.9
Targher, 2012	Italy	Cross‐sectional	90	47	26.7	0
Campello, 2014	Italy	Cross‐sectional study	60	47	32.2	50
Chitongo, 2017	UK	Cross‐sectional study	166	44	50.5	74
Gómez‐Ambrosi, 2006	Spain	Cross‐sectional	1089	42	37.4	66
Wildman, 2012	USA	Cross‐sectional	714	68	29	100
Plaisance, 2020	USA	Cross‐sectional	61	30	39 and 34	50
Meisel, 2020	Germany	Cross‐sectional	3366	52	34	52
Mangg, 2012	Austria	Prospective, observational study	206	33	27.8	71
Cazettes, 2012	USA	Cross‐sectional	63	58	31.4	48
Solà, 2012	Spain	Longitudinal, clinical intervention study	134	33	46	76
Nguyen, 2009	USA	Cross‐sectional	20,833	25	30–34.9	50
Tabassum, 2013	UK	Cross‐sectional	4289	55	33 and 34.7	60
Morcillo, 2023	Switzerland	Cross‐sectional	464	63	> 30	48
De Oliveira, 2015	Brazil	Cross‐sectional	196	47	35.1	75
Moreira, 2013	Brazil	Cross‐sectional	49	73	28.6	95
Bibi, 2014	Germany	Cross‐sectional	1222	50	32.1	25
Van Dongen, 2015	Netherlands	Longitudinal	348	38	28.4	69
Ilinčić, 2016	Serbia	Cross‐sectional	75	35	40.8	68
Bilooka, 2021	Ukraine	Cross‐sectional	31	32	31.1	70
Mariani, 2018	Italy	Cross‐sectional	101	41	41	75
Polac, 2003	Poland	Cross‐sectional	41	31	> 25	100
Samocha‐Bonet, 2012	Israel	Cross‐sectional	65	42	41	75
Calori, 2010	Italy	Population survey logitudinal	751	55	32.5	72
Strohacker, 2013	USA	Population survey	738	55	35.9	47
Hoekstra, 2014	USA	Population survey logitudinal	364	48	30.3	40
Rossi, 2020	Italy	Cross‐sectional	184	71	31.6	67
Espino, 2011	Chile	Cross‐sectional study	121	42	41	74
Lempesis, 2023	UK	Cross‐sectional study	15	56	34	100
Mohd Nor, 2018	Malaysia	Cross‐sectional	280	46	29	72
Pardina, 2012	Spain	Interventional	56	24	48.8	70
Taskiran, 2010	Turkey	Cross‐sectional	86	51	> 30	100
Villalpando, 2022	Mexico	Cross‐sectional	81	34	33.5	n.a.
Patel, 2016	UK	Cross‐sectional	100	42	27.4	50
Calderara, 2020	Switzerland	Cross‐sectional study	52	39	> 35	n.a.
De Laat‐Kremers, 2022	Italy	Cohort study	22,546	55	> 30	50
Balagopal, 2008	USA	Cross‐sectional study	13	16	99.1% tile	50
Semeraro, 2011	Italy	Cross‐sectional study	99	11	30.6	46
Stoppa‐Vaucher, 2012	Switzerland	Cross‐sectional	50	11	29	41
Giordano, 2011	Italy	Cross‐sectional	99	10	29.9	46
Singh, 2013	USA	Cross‐sectional	43	10	28.7	60
Dallar Bilge, 2012	Turkey	Cross‐sectional study	83	11	28.3	68
Tsiroukidou, 2021	Greece	Cross‐sectional	63	11	29.8	42
Mangg, 2012	Austria	Prospective, observational study	104	13	28	62
Halle, 2004	Germany	Cross‐sectional	197	12	25.9	54
Tirsi, 2014	USA	Cross‐sectional	102	17	37.8	66
Can, 2017	Turkey	Cross‐sectional	98	14	98.6% tile	65
Balagopal, 2002	USA	Cross‐sectional	12	16	36.6	100
Pérez, 2015	Puerto Rico	Cross‐sectional	101	15	30.7	44
Akinci, 2008	Turkey	Cross‐sectional	41	10	21.8	50
Valle Jimenez, 2007	Spain	Case–control study	92	7	23.7	58
Fritsch, 2010	Germany	Case–control study	90	12	27.4	50
Meyer, 2006	Germany	Case–control study	52	15	30.6	53
Olza, 2014	Spain	Case–control study	446	9	27	46
Cura‐Esquivel, 2023	Mexico	Cross‐sectional analytical study	43	9	27	36
Rupérez, 2018	Spain	Cross‐sectional analytical study	384	8	> 25	50
Roberts, 2013	USA	Cross‐sectional	33	13	33	60
Mantovani, 2011	Brazil	Cross‐sectional	86	10	98.2% tile	50
Wei, 2013	China	Cross‐sectional	60	11	> 30	50
Cimenti, 2006	Austria	Prospective	26	10	33.2	30
Siklar, 2011	Turkey	Cross‐sectional study	158	11	26.9	72

All studies included in the meta‐analysis were characterized regarding their study design, country of origin, number of participants, gender distribution, and age (Table 1). The overall risk of bias is considered maximal moderate, as indicated by the high NOS values throughout the studies (Table 2).

**TABLE 2:** The quality of the studies defined by NOS scale.

Study	Study design	Is the case definition adequate?	Representativeness of the cases	Selection of controls	Definition of controls	Comparability of cases and controls on the basis of the design or analysis for Age and Sex	Ascertainment of exposure	Same method of ascertainment for cases and controls	Non‐Response rate	Quality score
		max 1 star	max 1 star	max 1 star	max 1 star	max 2 star	max 1 star	max 1 star	max 1 star	max 9 star
P Balagopal, 2008	cross‐sectional	1	1	B	1	2	1	1	C	7
Agnieszka Firszt‐Adamczyk, 2006	cross‐sectional	1	1	B	1	2	1	1	C	7
Fabrizio Semeraro, 2011	cross‐sectional	1	1	B	1	2	1	1	C	7
Sophie Stoppa‐Vaucher, 2012	cross‐sectional	1	1	B	1	2	1	1	C	7
Anamika Singh, 2013	Cross‐Sectional Study	1	1	C	1	1	1	1	C	6
Sanna M. Kaye,2012	population‐based, longitudinal study, multicenter	1	1	1	1	2	1	1	1	9
Giovanni Targher, 2012	cross‐sectional study	1	1	1	1	2	1	1	C	8
Elena Campello, 2014	cross‐sectional study	1	1	B	1	2	1	1	C	7
P B Chitongo,2017	cross‐sectional study	1	1	1	1	2	1	1	C	8
Yildiz Dallar Bilge, 2012	cross‐sectional study	1	1	1	1	1	1	1	C	7
Javier Gómez‐Ambrosi, 2006	cross‐sectional study	1	1	1	1	2	1	1	C	8
RP Wildman, 2012	cross‐sectional study	1	1	1	1	2	1	1	1	9
Eric P Plaisance, 2020	cross‐sectional study	1	1	1	1	2	1	1	C	8
Kyriaki Tsiroukidou, 2021	cross‐sectional study	1	1	1	1	2	1	1	C	8
Meisel P, 2020	cross‐sectional study	1	1	1	B	2	1	1	1	8
Harald Mangg, 2012	cohort study (longitudinal)	1	1	1	1	2	1	1	C	8
Fanny Cazettes, 2011	cross‐sectional study	1	1	1	1	2	1	1	C	8
Eva Solá, 2012	cross‐sectional study + intervention	1	1	1	1	2	1	1	C	8
Xuan‐Mai T Nguyen, 2009	cross‐sectional study	1	1	1	B	1	1	1	C	6
Faiza Tabassum,2013	cross‐sectional study	1	1	1	B	2	1	1	C	7
Maximilian Iglesias Morcillo,2023	cross‐sectional study	1	1	1	B	1	1	1	1	7
Raquel de Oliveira,2015	cross‐sectional study	1	1	B	1	1	1	1	C	6
P.F.P. Moreira,2013	cross‐sectional study	1	1	1	B	1	1	1	C	6
Saima Bibi,2024	cohort study (longitudinal)	1	1	1	1	0	1	1	C	6
Jenny van Dongen,2015	cohort study (longitudinal)	1	1	1	1	2	1	1	C	8
Branislava Ilinčić,2016	cross‐sectional study	1	1	B	1	1	1	1	C	6
Yuliya Vyacheslavivna Bilooka ,2021	cross‐sectional study	1	1	C	1	2	1	1	C	7
Martin Halle, 2004	cross‐sectional study	1	1	1	1	1	1	1	C	7
Stefania Mariani, 2018	cross‐sectional study	1	1	B	1	2	1	1	C	7
Aziz Tirsi, 2014	cross‐sectional study	1	1	1	1	1	1	1	C	7
Eva Pardina, 2012	cross‐sectional study	1	1	B	1	1	1	1	C	6
I. Polac, 2003	cross‐sectional study	1	1	C	1	2	1	1	C	7
Ummugulsum Can, 2017	cross‐sectional study	1	1	1	1	2	1	1	C	8
Dorit Samocha‐Bonet, 2012	cross‐sectional study	1	1	C	B	2	1	1	C	6
Giliola Calori, 2010	cohort study (longitudinal)	1	1	1	1	1	1	1	C	7
Prabhakaran Balagopal, 2002	cross‐sectional study	1	1	B	B	2	1	1	C	6
Cynthia M Pérez, 2015	cross‐sectional study	1	1	B	B	2	1	1	C	6
Gulcin Akinci, 2008	cross‐sectional study	1	1	C	B	2	1	1	C	6
Kelley Strohacker, 2013	cross‐sectional study	1	1	1	B	2	1	1	C	7
Trynke Hoekstra, 2014	cohort study (longitudinal)	1	1	1	1	2	1	1	C	8
Andrea P Rossi, 2020	cross‐sectional study	1	1	1	1	2	1	1	1	9
Paola Giordano, 2011	cross‐sectional study	1	1	1	1	1	1	1	C	7
Miguel Valle Jimenez, 2007	case‐control Study	1	1	C	1	2	1	1	C	7
P. Fritsch, 2010	cross‐sectional study	1	1	B	1	2	1	1	C	7
Andreas Alexander Meyer, 2006	cross‐sectional study	1	1	1	1	2	1	1	C	8
Ceo, 2012	cross‐sectional study	1	1	1	1	1	1	1	C	7
Małgorzata Michalska,2013	cross‐sectional study	1	1	C	1	1	1	1	C	6
Espino, 2011	cross‐sectional study	1	1	1	1	2	1	1	C	8
Ioannis G Lempesis, 2023	cross‐sectional study	1	1	C	B	2	1	1	C	6
Cura–Esquivel, 2023	cross‐sectional study	1	1	B	1	1	1	1	C	6
A.I. Rupérez, 2018	cross‐sectional study	1	1	1	1	1	1	1	C	7
Noor Shafina Mohd Nor, 2018	cross‐sectional study	1	1	1	B	2	1	1	C	7
Olza, 2014	case–control, multicentre study	1	1	1	1	2	1	1	C	8
Roberts, 2023	interventional study	1	1	C	1	2	1	1	C	7
Mantovani, 2011	cross‐sectional study	1	1	C	1	2	1	1	C	7
Ying Wei, 2013	cross‐sectional study	1	1	B	1	2	1	1	C	7
Diana Carolina Villalpando Sánchez, 2022	cross‐sectional study	1	1	C	1	1	1	1	1	7
Patel, 2016	cross‐sectional study	1	1	B	1	2	1	1	C	7
Calderara,2020	cross‐sectional study	1	1	C	1	1	1	1	C	6
de Laat‐Kremers,2022	cohort study	1	1	1	1	1	1	1	1	8
Cimenti, 2006	prospective study	1	1	C	1	2	1	1	C	7
Siklar, 2011	cross‐sectional study	1	1	C	1	2	1	1	C	7
Taskiran, 2010	cross‐sectional study	1	1	C	1	2	1	1	C	7

[Correction added on 4 May 2026, after first online publication: Table 2 has been updated in this version.]

*Note:* Study quality was assessed using the Newcastle–Ottawa Scale (NOS), adapted for cohort, case–control and cross‐sectional studies. The NOS evaluates three domains (selection, comparability, and exposure/outcome), with a maximum of nine stars; higher scores indicate lower risk of bias. Up to two stars were awarded for comparability, reflecting adjustment for age and for sex or other relevant confounders. At the item level, “1” indicates that a criterion was fulfilled (star awarded), whereas “B” or “C” indicate that the criterion was not met. Additional coding was applied for selected items: For selection of controls, “B” denotes hospital controls, and “C” indicates no description; for definition of controls, “B” indicates that the source was not described; for non‐response rate, “C” indicates that response rates were not reported or that non‐response bias was not addressed.

The included studies comprised overall 59,503 patients (56,992 adults and 2511 children) with obesity or normal weight. The mean age of the adults was 44.7 years ranging from 24 to 73 years. The mean age of children in included studies was 11.7 years and well comparable (*p* = 0.82, Figure S1). Studies were conducted in Europe (*n* = 37), North America (*n* = 11), South America (*n* = 8), and Asia (*n* = 8).

### 3.2 Levels of Coagulation Activation Parameters in Obesity

In 64 studies with overall 59,503 patients, the plasma levels of biomarkers of coagulation activation D‐dimer, fibrinogen, PAI‐1, vWF (antigen concentration), FVIII activity, or ETP were analyzed and compared with controls in children or adults. When applicable, subgroup analysis for gender‐specific effects was performed.

**FIGURE 2:**
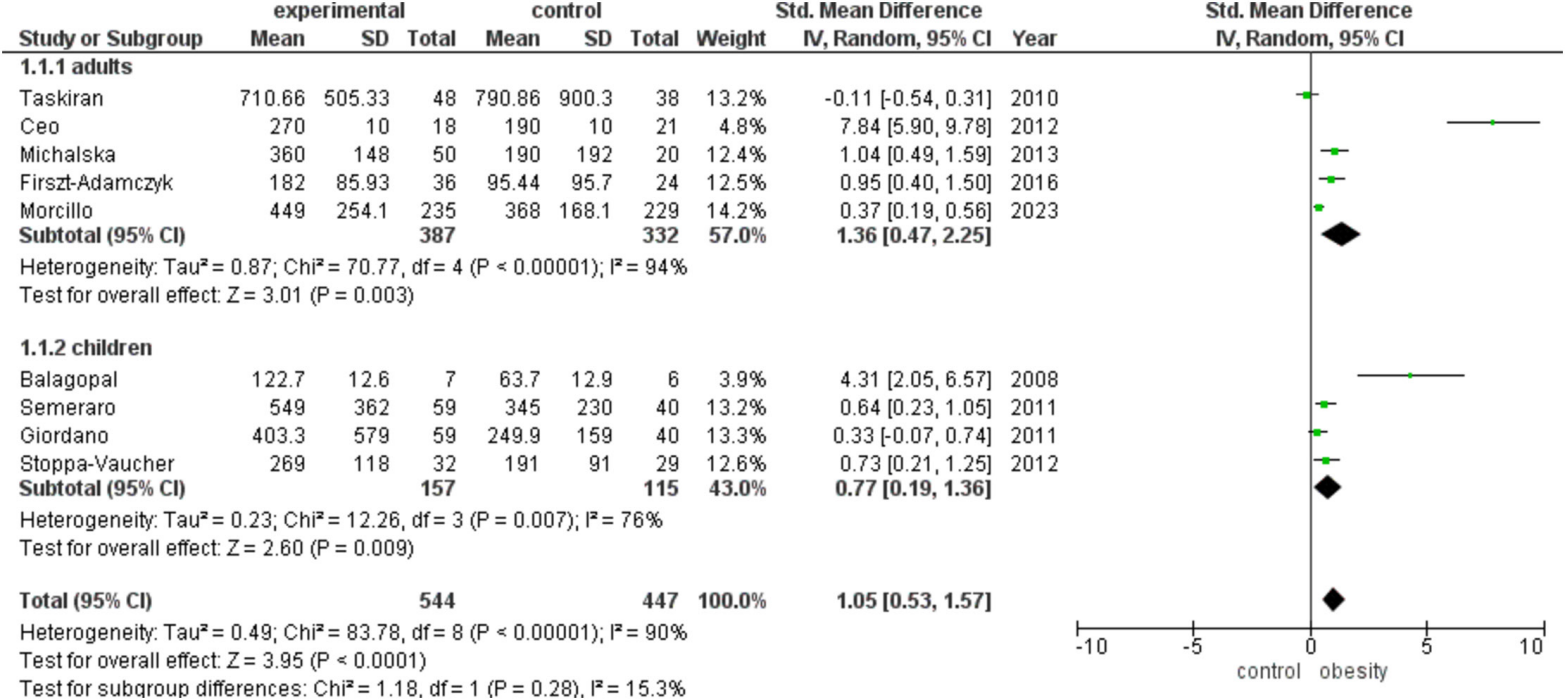
Forest plot of the meta‐analysis of standardized mean difference of D‐dimer levels by study group (obesity vs. control group). CI, confidence interval, df, degrees of freedom; SD, standard deviation.

D‐dimer levels were increased in adults (SMD 1.36, 95% CI 0.47–2.25, *p* = 0.003, Figure 2) and in children (SMD 0.77, 95% CI 0.19–1.36, *p* = 0.009, Figure 2). When combining all 8 studies, D‐dimer levels were elevated (SMD 1.05, 95% CI 0.53–1.57, *p* < 0.0001, Figure 2). The effect of obesity observed in children versus adults indicates an at least partially different interaction of obesity with the coagulation and inflammation system according to age. Ceo 2012 is the only study in the adults which showed a clear increase of D‐dimer levels and also a study solely investigating females in Brazil with a small cohort size [70]. A low number of published manuscripts, especially targeting the gender‐specific aspect, was available. The heterogeneity among the included studies was overall moderate to high in the groups assessing D‐dimer levels (*I* ^2^ = 90%).

**FIGURE 3:**
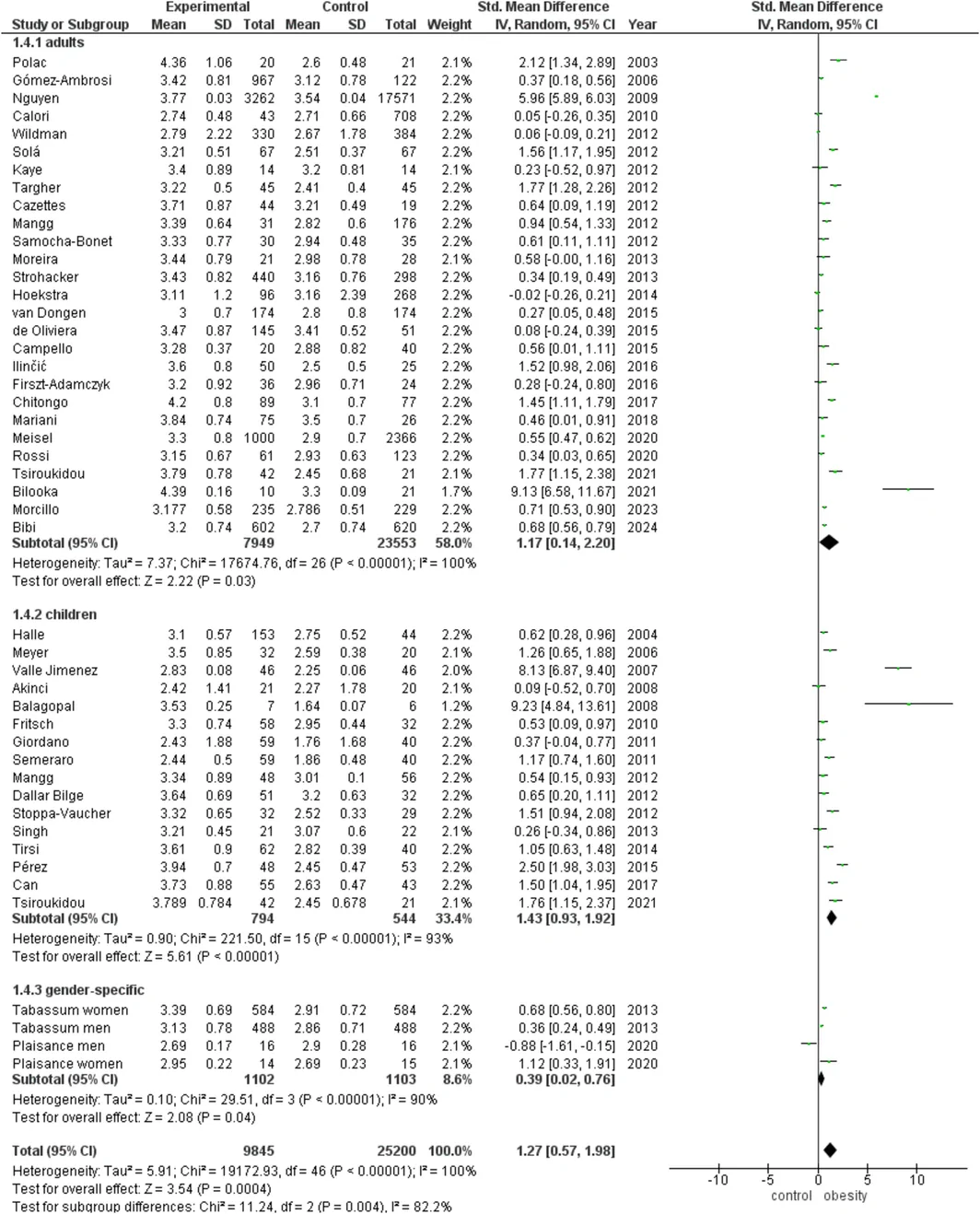
Forest plot of the meta‐analysis of standardized mean difference of fibrinogen by study group (obesity vs. control group). CI, confidence interval, df, degrees of freedom; SD, standard deviation.

Like D‐dimer, fibrinogen is associated with inflammation and thrombosis [87]. As fibrinogen is a precursor of plasma D‐dimer, we next determined fibrinogen levels in relation to obesity. In the pooled analyses of 43 studies assessing fibrinogen, fibrinogen levels were increased in adults (SMD 1.17, 95% CI 0.14–2.20, *p* = 0.03, Figure 3) and to a larger extend in children (SMD 1.43, 95% CI 0.93–1.92, *p* < 0.0001, Figure 3), resulting in a total SMD of 1.27 (95% CI 0.57–1.98, *p* < 0.0001, Figure 3). There were no clear outliers among studies in this dataset. Gender‐specific subanalysis reveals that women in the two studies differentiating data according to sex have higher fibrinogen values (SMD = 0.39; 95% CI 0.02–0.76, *p* = 0.04, Figure 3) [36, 44]. Notable in Plaisance's study, men with obesity had lower fibrinogen than their control group [36]—a finding not observed by others. The heterogeneity among the included studies was high in the groups assessing fibrinogen levels (*I* ^2^ = 100%).

**FIGURE 4:**
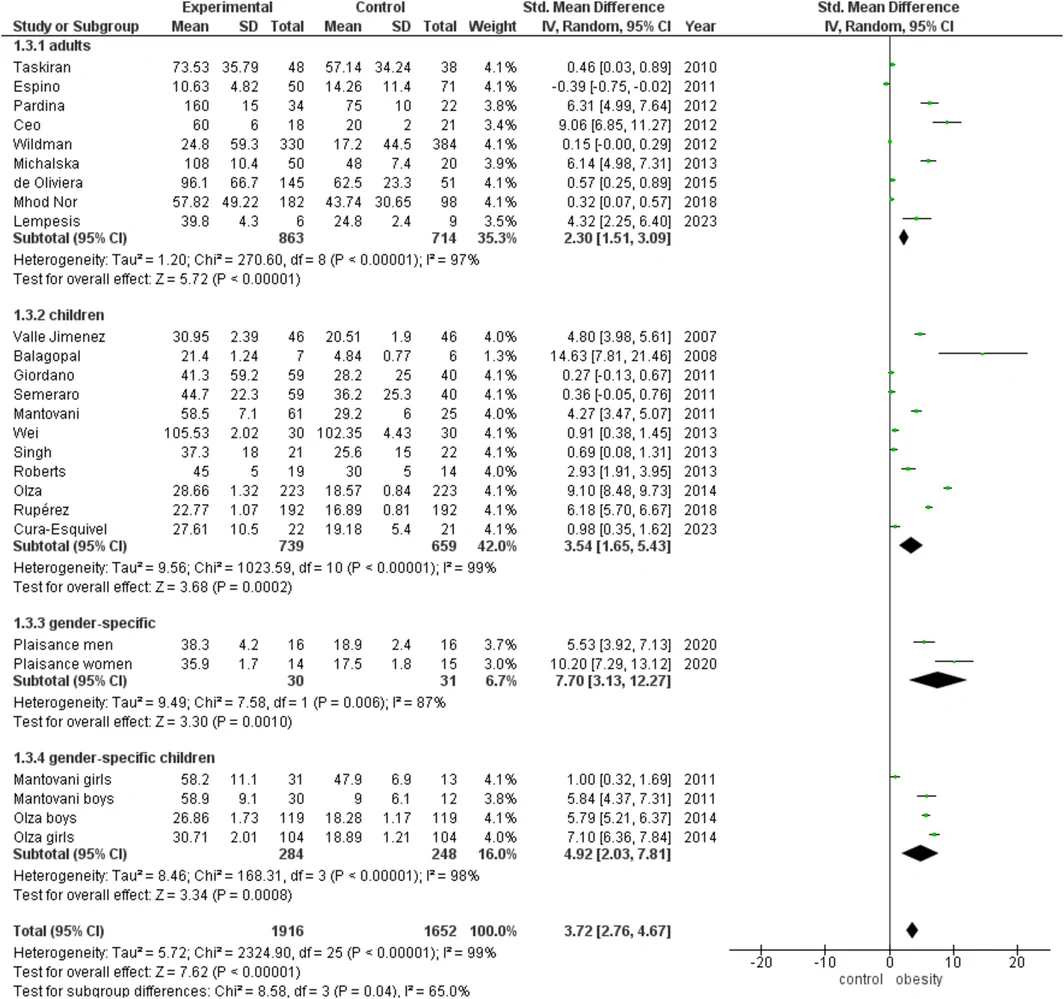
Forest plot of the meta‐analysis of standardized mean difference of PAI‐1 by study group (obesity vs. control group). CI, confidence interval; df, degrees of freedom; PAI‐1, plasminogen activator inhibitor 1; SD, standard deviation.

Another factor regulating D‐dimer is fibrinolysis. In the current meta‐analysis, 20 studies reporting PAI‐1 levels in individuals with obesity were included. PAI‐1 was increased in eight of nine studies including adults, resulting in an SMD of 2.30 (95% CI 1.51–3.09, *p* < 0.0001, Figure 4). In 11 studies including children, PAI‐1 levels were tentatively increased in 2 and significantly increased in 9 studies, resulting overall in an SMD of 3.54 (95% CI 1.65–5.43, *p* = 0.0002, Figure 4). When combining adults and children, the SMD was significantly increased across studies (SMD = 3.75; 95% CI 2.75–4.75, *p* < 0.0001, Figure 4). Notably, three studies including adults, which could be included, assessed only females. Gender‐specific subanalysis showed increased PAI‐1 levels in females (SMD = 10.2; 95% CI 7.29–13.12 vs. SDM = 5.53; 95% CI 3.92–7.13, Figure 4) as compared with men (*n* = 1 study). Targeting children, studies revealed controversial effects: Although one study describing PAI‐1 levels in girls with obesity indicated higher, yet the second study lower PAI‐1 levels in girls as compared with boys (Figure 4, *n* = 2 studies). The heterogeneity among the included studies was high in the groups assessing PAI‐1 levels (*I* ^2^ = 99%).

**FIGURE 5:**
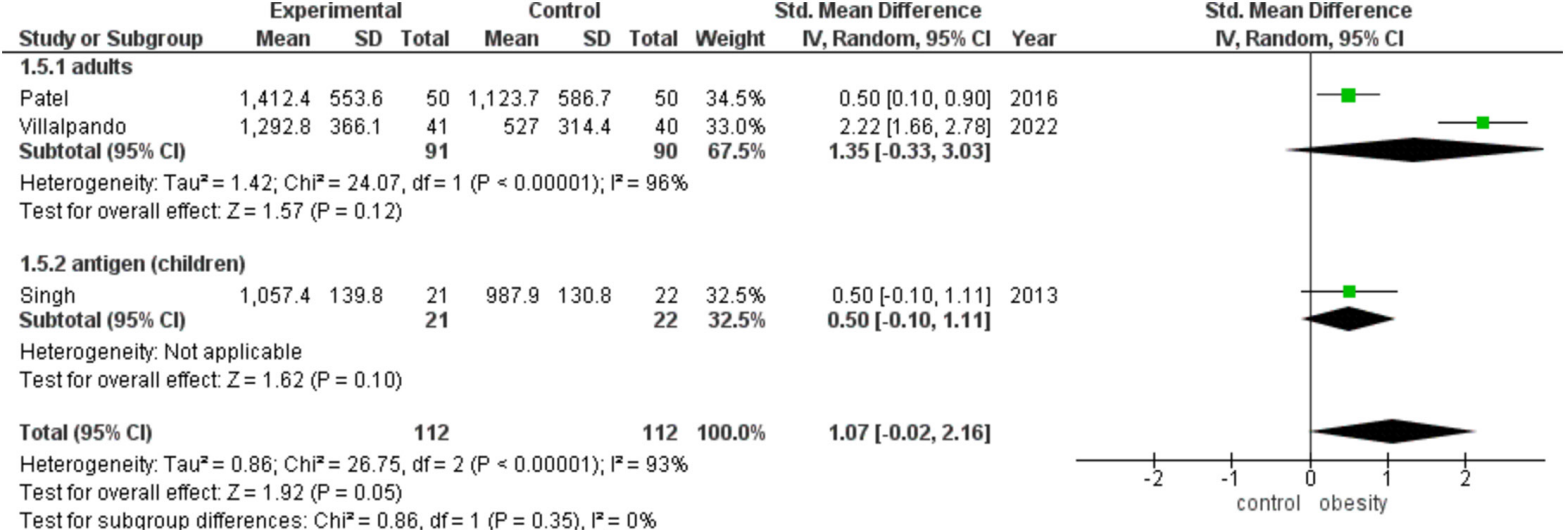
Forest plot of the meta‐analysis of standardized mean difference of vWF antigen by study group (obesity vs. control group). CI, confidence interval, df, degrees of freedom; SD, standard deviation; vWF, von Willebrand factor.

FVIII and vWF are both coagulation factors promoting clot formation, thus indirectly contributing to D‐dimer. The data of one study involving vWF levels (antigen) in children was included, revealing an SMD of 0.50 (95% CI −0.10, 1.11, *p* = 0.1, Figure 5). When analyzing the two studies including adults, vWF antigen showed an SMD of 1.35 (95% CI −0.33, 3.03, *p* = 0.12, Figure 5), reflecting a tendency of increased vWF values in obesity. Importantly, the two studies individually reported higher levels of vWF in adults with obesity [88], but when combining the two studies, the heterogeneity resulted in only tentatively increased vWF levels.

**FIGURE 6:**
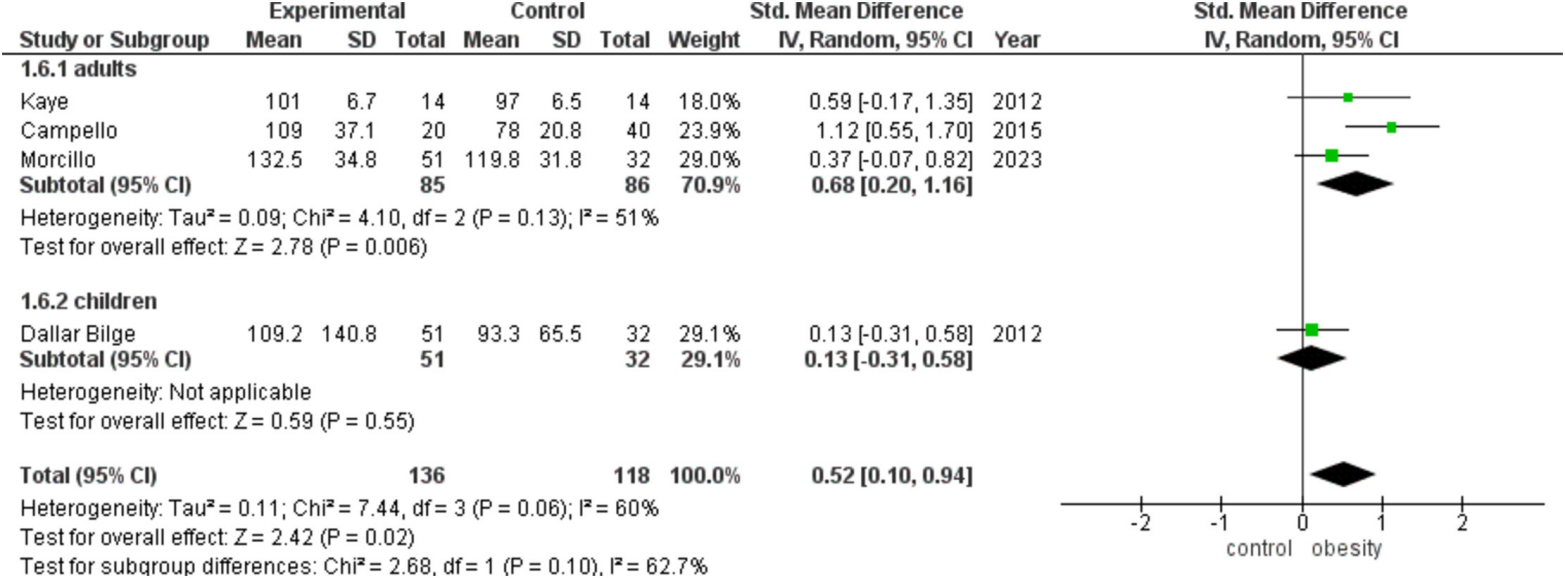
Forest plot of the meta‐analysis of standardized mean difference of FVIII activity by study group (obesity vs. control group). CI, confidence interval, df, degrees of freedom; SD, standard deviation.

Assessing high FVIII activity has been linked to obesity and insulin resistance [89]. Corroborating this, the total SMD of FVIII activity in obesity was 0.52 (95% CI 0.10–0.94, *p* = 0.02, Figure 6), comprising the pooled data of three studies with adults and one study with children. Notably, of the four included studies, only one showed a clear SMD of higher FVIII activity in obesity [33]. Both vWF and FVIII reported in the included studies did not show a clear trend of higher levels in obesity. The heterogeneity among the included studies was overall moderate to high in the groups assessing vWF antigen (*I* ^2^ = 93%) and FVIII (*I* ^2^ = 60%).

**FIGURE 7:**
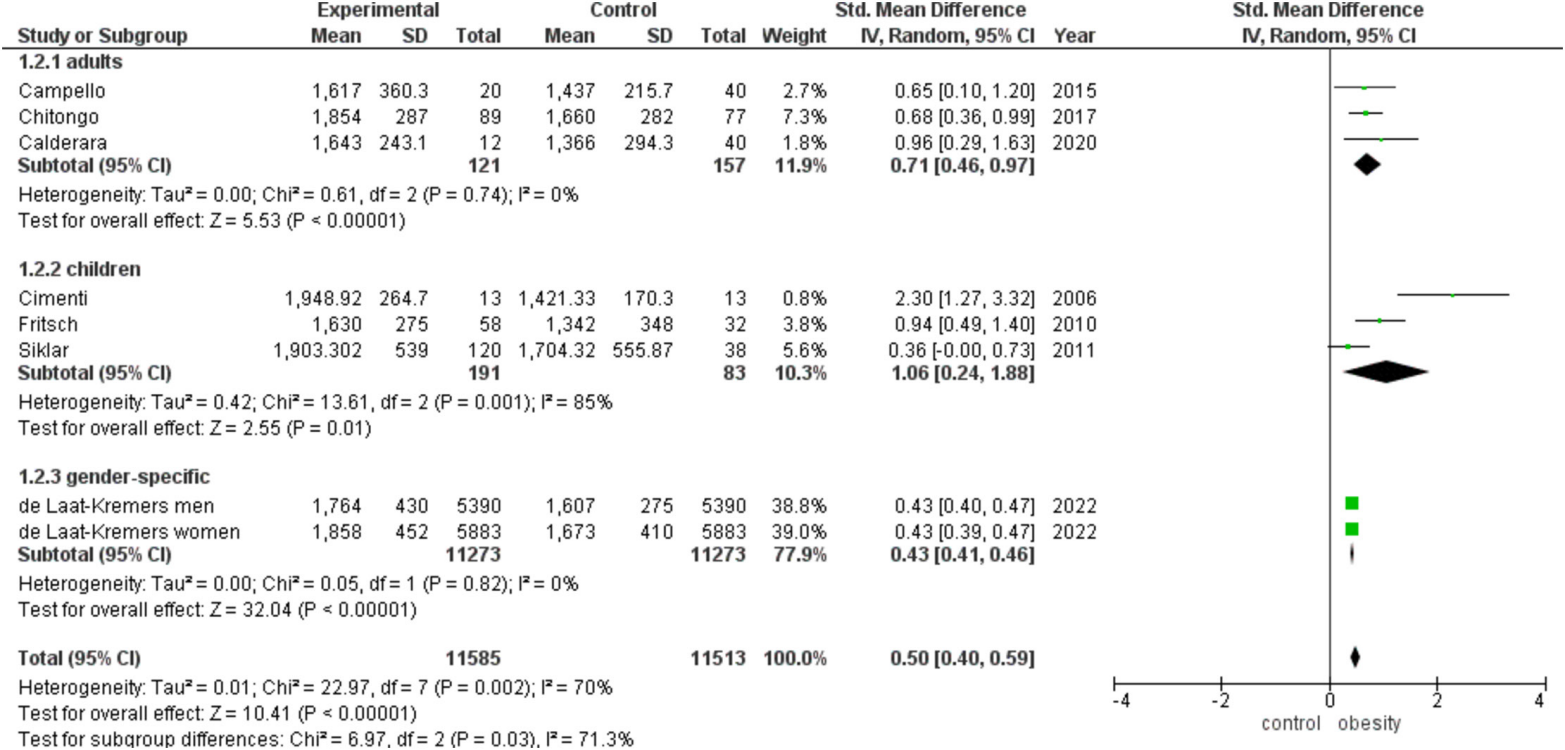
Forest plot of the meta‐analysis of standardized mean difference of ETP by study group (obesity vs. control group). CI, confidence interval; df, degrees of freedom; ETP, endogenous thrombin potential; SD, standard deviation.

FVIII and vWF promote thrombin generation. ETP levels were increased in all seven included studies involving adults and children, with an SMD of 0.50 (95% CI 0.40–0.59, *p* < 0.001, Figure 7). In children, the SDM was 1.06 (95% CI 0.24–1.88, *p* = 0.01, Figure 7), and in adults, the SDM was 0.71 (95% CI 0.46–0.97, *p* < 0.0001, Figure 7). The heterogeneity among the included studies was moderate in the groups assessing ETP (*I* ^2^ = 70%).

Despite this heterogeneity, the results overall indicate coagulation activation in obesity, irrespective of age. The effect sizes were relatively homogeneous. The only exception was the analyses of fibrinogen levels by Plaisance, which showed lower levels in males with obesity compared with controls. Although the study numbers in the subgroups (children and gender‐specific) were rather small (1–6 studies), the pooling of the studies shows a consistent trend, and the combined effect was significant for all markers, with an increase of the pooled SMD for each marker of coagulation activation. Although some adult studies showed variability (such as Ceo 2012 in D‐dimer levels), the overall trends remained robust. The total effect sizes for each parameter were statistically significant, further supporting the association between obesity and a pro‐thrombotic state. Of note, the association of increased coagulation activation markers with obesity was particularly pronounced in children. Collectively, obesity is associated with a higher degree of coagulation activation, as reflected by increased levels of D‐dimer, fibrinogen, PAI‐1, vWF, FVIII, and ETP.

### 3.3 Risk of Publication Bias

Publication bias was assessed using funnel plots and Egger's regression test when at least 10 studies were available. Funnel plots for fibrinogen and PAI‐1 values are shown in Figure S2–S4. For fibrinogen in adults (*n* = 27), Egger's regression test indicated evidence of funnel plot asymmetry (*p* < 0.001, Figure S2). Egger's regression indicates statistically significant funnel plot asymmetry, consistent with small‐study effects in adult studies of fibrinogen. If we removed the two clear outliners, results remain significant. For fibrinogen in children (*n* = 16), Egger's regression test did not indicate funnel plot asymmetry (*p* = 0.21, Figure S3). For PAI‐1 outcome (children only; *n* = 11, Figure S4), Egger's test did not indicate funnel plot asymmetry (*p* = 0.99). For the remaining parameters, the small number of studies in both adults and children precluded a meaningful statistical assessment of publication bias.

### 3.4 Sensitivity Analysis

Due to methodological heterogeneities, sensitivity analyses were applied to figures for parameters fibrinogen, PAI‐1 and D‐dimer. The other parameters were homogenous regarding methods used. For Figures 2, 3, 4, sensitivity analyses were performed and added to Figures S5–S8. In brief, alternative methods (Table S1) were excluded, then forest plots generated and pooled effects were compared with the original analysis. For D‐dimer, sensitivity analysis revealed consistent SMDs comparable with the initial analysis, with an overall SDM of 1.49 (95% CI 0.67–2.31, *p* = 0.0004, Figure S5). PAI‐1 remained significantly increased in adults and children, resulting overall in an SMD of 3.40 (95% CI 2.55–4.25, *p* < 0.0001, Figure S6). Although fibrinogen levels remained increased in children and adults after removal of alternative methods (Figure S7), the effect in adults was not significant after further removal of all publications stating “n.a.” regarding method used (Figure S8). Nevertheless, the pooled effect remained significant in all cases.

## 4 Discussion

Obesity is a chronic low‐grade inflammatory and hypercoagulable condition, which is associated with an increased risk of cardiovascular and thrombotic events. This meta‐analysis demonstrates consistently elevated markers of coagulation activation in obesity, including D‐dimer, fibrinogen, PAI‐1, vWF, FVIII, and ETP. Together, these findings underscore the prothrombotic phenotype of obesity and point to potential underlying mechanisms, such as systemic inflammation and endothelial dysfunction. Importantly, several coagulation markers exhibited age‐dependent differences between children and adults.

The more pronounced increase in fibrinogen levels in children compared with adults, in combination with the relatively smaller increase in D‐dimer levels, suggests age‐dependent differences in D‐dimer dynamics between children and adults. The distinct biomarker patterns observed across age groups may reflect age‐specific regulatory mechanisms governing fibrinogen turnover. Previous studies indicate that increased fibrinogen concentrations in children with obesity are primarily driven by an enhanced fractional synthesis rates [26], whereas the comparatively elevated fibrinogen levels in adult and elderly populations appear to be more strongly influenced by reduced breakdown or clearance [90]. Developmental differences in hepatic function and protein turnover may therefore contribute to the divergent fibrinogen and D‐dimer profiles observed across age groups and should be considered when interpreting biomarker elevations in pediatric populations. Although direct quantitative comparisons between age groups are limited by heterogeneity among studies, including differences in analytical methods, consideration of this variability within the groups themselves provides important mechanistic insights. Notably, comparison of children and adults suggests that increased fibrinogen levels do not necessarily translate into proportional increases in D‐dimer levels. Other factors, such as inflammatory activity or differences in hepatic fibrinogen synthesis, may differentially affect fibrinogen and D‐dimer concentrations in children and adults. Possible explanations for the disproportionately higher fibrinogen levels observed in children with obesity, in the absence of similarly increased D‐dimer levels, include reduced clot formation or age‐specific differences in fibrin removal pathways. Indeed, higher PAI‐1 levels in children suggest reduced fibrinolytic activity, which would be expected to result in lower D‐dimer generation. Alternative, plasmin‐independent pathways of fibrin degradation have been proposed [91]; however, whether such mechanisms play a relevant role in pediatric populations remains to be determined. Although total fibrinogen was the focus of the present meta‐analysis, emerging evidence suggests that γ′ (gamma‐prime) fibrinogen may represent a more specific marker of thrombo‐inflammatory risk, particularly in obesity and inflammatory states [92, 93, 94, 95]. However, the current evidence base is limited, and available studies are methodologically heterogeneous and frequently lack appropriate control data, precluding quantitative synthesis. Future studies employing standardized measurement and reporting of γ′ fibrinogen are therefore warranted to clarify its potential role and enable meta‐analytic evaluation.

Fibrin removal is a critical process for maintaining hemostatic balance, and while the core fibrinolytic pathways are largely conserved between adults and children, developmental differences exist in the regulation, efficiency, and activity of key components. PAI‐1 levels are typically higher in neonates, which can result in relatively reduced fibrinolytic activity. Plasminogen concentrations are lower in neonates but gradually reach adult levels during childhood. Similarly, levels of the plasminogen activators tPA and uPA are generally lower in neonates and infants than in adults [96]. α2‐antiplasmin levels are also reduced in neonates, thereby affecting the regulation of fibrinolysis [97]. Collectively, these features contribute to a hypofibrinolytic state in neonates and infants, which is consistent with the more pronounced increase in PAI‐1 observed in younger individuals. However, the reasons why PAI‐1 levels increase more strongly in children than in adults, and the extent to which adipose tissue contributes to this effect, remain to be elucidated.

One of the most striking findings of our meta‐analysis was the pronounced elevation of PAI‐1 levels in individuals with obesity, particularly in children. PAI‐1, an important adipokine, is well recognized for its pro‐inflammatory properties [98]. As a potent inhibitor of fibrinolysis that is predominantly secreted by adipose tissue, PAI‐1 represents a key mediator of obesity‐associated disturbances in hemostasis. Over the past two decades, clinical studies have consistently demonstrated an association between obesity and impaired fibrinolysis [9, 99]. Excess adipose tissue contributes to increased PAI‐1 production, thereby disrupting fibrinolytic balance [100]. As noted previously, PAI‐1 is partly produced and released by human adipose tissue [98, 101], and circulating levels correlate with measures of adiposity and features of the metabolic syndrome [102]. Adipocytes from individuals with obesity secrete approximately twice as much PAI‐1 as those from lean individuals [103]. Moreover, elevated PAI‐1 levels are particularly pronounced in subjects with abdominal obesity [9, 104]. In this context, obesity‐associated inflammation may further exacerbate coagulation activation, as pro‐inflammatory cytokines promote fibrinogen synthesis, endothelial activation, and dysregulation of fibrinolytic pathways, including increased PAI‐1 expression [105]. Together, these findings indicate that, beyond enhanced coagulation activity, obesity‐related alterations in fibrinolysis may contribute to the prothrombotic phenotype observed in obesity.

The current meta‐analysis reveals that childhood obesity is characterized not only by increased fibrinogen and PAI‐1 levels but also by elevated endogenous thrombin potential (ETP), which was nominally more pronounced than in adults. This heightened coagulation response in children may be explained by developmental differences in hemostatic regulation. Pediatric hemostasis differs fundamentally from that of adults, with physiologically lower levels of anticoagulant proteins and a greater dependence on thrombin generation for clot stability, particularly during newborn and early childhood stages [106]. When combined with the pro‐inflammatory milieu associated with obesity, these physiological characteristics may amplify coagulation activation in pediatric populations.

It is well established that the clinical risk of thrombosis increases with age, and that genetic or acquired risk factors, such as factor V Leiden (FVL) or obesity, often clinically manifest later in life [107, 108]. The present findings demonstrate that coagulation activation is already detectable in children with obesity, although it may not yet be clinically “unmasked” until adulthood. Taken together, these observations support the concept that obesity during childhood may confer an increased risk of thrombotic disease later in life [109, 110].

Obesity is a chronic low‐grade inflammatory state [111] and is associated with increased levels of pro‐inflammatory cytokines [112]. All biomarkers assessed in the present meta‐analysis are, to varying degrees, linked to inflammatory processes, which are commonly observed in obesity [111, 113]. Accordingly, elevated levels of D‐dimer, fibrinogen, PAI‐1, vWF, and FVIII may, at least in part, reflect the chronic low‐grade inflammation associated with obesity [113, 114]. Consistent with this concept, D‐dimer and vWF are known to be increased in chronic inflammatory disease states [115, 116, 117]. Low‐grade inflammation is more pronounced in women with obesity than in men, as reflected by higher levels of C‐reactive protein (CRP) and fibrinogen [37, 118]. In the limited number of studies available, the parameters fibrinogen, ETP, and PAI‐1 consistently showed trends toward higher levels in women (or girls) compared with men (or boys). This reciprocal interaction between inflammation and coagulation may therefore contribute to the increased coagulability observed in females with obesity, which could partly explain the higher risk of arterial and venous thrombotic events reported in this population. Beyond inflammation‐driven mechanisms, adipose tissue itself may directly contribute to coagulation activation. Adipocytes from individuals with obesity have been shown to produce coagulation factor VII (FVII) [119], and in mice fed a high‐fat diet, expression of tissue factor and factor VII is increased in adipose tissue [120]. These findings suggest that adipocytes may modulate coagulation activity independently of systemic inflammation by expressing and secreting coagulation factors.

In line with this concept, factor XIII (FXIII), a coagulation factor not typically associated with inflammation, appears to be increased in obesity. However, only two studies investigating FXIII in individuals with obesity were identified, one of which did not meet the inclusion criteria. In the remaining study, Pedersen et al. reported significantly elevated FXIII levels in individuals with obesity prior to bariatric surgery, with levels decreasing after weight loss [121]. Corroborating these findings, Kaye et al. examined monozygotic twins discordant for obesity and observed higher FXIII levels in the twin with obesity [31]. Although evidence is limited, these observations suggest that obesity‐associated hypercoagulability may not be solely secondary to low‐grade inflammation but may also involve inflammation‐independent alterations in coagulation factor regulation.

Endothelial dysfunction is a central feature of obesity, linking metabolic disturbances with vascular complications. The endothelium plays a crucial role in the regulation of hemostasis, in part through the release of vWF upon activation. In line with this, the present meta‐analysis identified elevated vWF levels in individuals with obesity, suggesting impaired endothelial function and compromised vascular homeostasis. Multiple obesity‐related factors, including oxidative stress, reduced nitric oxide bioavailability, elevated free fatty acids, and pro‐inflammatory adipokines, are known to contribute to endothelial dysfunction. In addition, hyperglycemia and insulin resistance have been shown to enhance endothelial vWF secretion, thereby reinforcing the interplay between metabolic dysregulation, endothelial dysfunction, and coagulation activation. Beyond vWF, dysregulation of other endothelial anticoagulant pathways, such as thrombomodulin and the endothelial protein C receptor (EPCR), may further contribute to obesity‐associated hypercoagulability [122].

Despite the overall robustness of this meta‐analysis, several limitations should be acknowledged. Substantial heterogeneity across studies, particularly for vWF and FVIII, suggests that additional modifiers, such as sex, ethnicity, and comorbid conditions, may influence coagulation activation in obesity. Given the moderate to high heterogeneity (*I* ^2^) observed across outcomes, random‐effects models were applied throughout the analyses. Although SMDs were consistently reported together with 95% CIs, the precision of effect estimates varied across outcomes and subgroups, as reflected by wide CIs and high heterogeneity for several parameters. This variability, especially in subgroup analyses with limited numbers of studies, should be considered when interpreting the magnitude and reliability of pooled effect sizes. In addition, many of the included studies were cross‐sectional or retrospective in design, which precludes causal inference. Accordingly, the observed associations between obesity and biomarker elevations should be interpreted as correlational rather than causal, and longitudinal or prospective studies are required to clarify temporal relationships and underlying causal pathways. Consistent with the American Heart Association Scientific Statement [19], the biomarkers assessed in this study reflect underlying inflammatory and hemostatic pathways and should be interpreted as indicators of pathophysiological processes rather than as isolated risk predictors. Among the parameters analyzed, fibrinogen, PAI‐1, and D‐dimer have been discussed as emerging biomarkers in pediatric populations. However, the concept of novel biomarkers should not be restricted to thrombotic risk alone, but should be expanded to encompass related processes such as inflammation, oxidative stress, insulin resistance, and adipocyte dysfunction, as emphasized previously [19]. The elevation of coagulation markers observed in obesity raises several clinically relevant considerations. Although our findings indicate an association between obesity and a hypercoagulable state, it remains uncertain whether these biomarker alterations translate into an increased incidence of thrombotic events. Most of the included studies were retrospective in design and involved relatively small sample sizes, which limits causal interpretation. In addition, heterogeneity in laboratory assays used to measure coagulation parameters likely contributed to variability across studies. Sensitivity analyses demonstrated that the main findings for D‐dimer and PAI‐1 were robust to the exclusion of studies using alternative measurement methods. In contrast, for fibrinogen, attenuation of the effect in adults following further restriction to studies with clearly reported methods suggests that methodological heterogeneity may partially account for variability in effect estimates; nevertheless, the overall direction of the association remained consistent.

Evidence of funnel plot asymmetry for fibrinogen in adults suggests that small‐study effects cannot be excluded, whereas the absence of asymmetry for other outcomes and age groups supports the overall robustness of the findings, albeit with limited statistical power for some parameters. In addition, only studies published in the English language were included to ensure clarity and consistency of data extraction. Consequently, relevant studies published in other languages may not have been captured and could not be included in the present analysis.

Future research should prioritize longitudinal studies to determine whether obesity‐associated coagulation abnormalities persist over time and predict clinically relevant outcomes. Several studies have demonstrated that lifestyle‐induced or surgically induced weight loss is accompanied by a reduction in coagulation activation, suggesting a degree of reversibility of these alterations. However, further studies are required to clarify whether such improvements translate into a reduced incidence of thromboembolic events and whether novel pharmacological weight‐loss interventions, such as GLP‐1 receptor agonists or combined GLP‐1/GIP agonists, exert comparable beneficial effects on coagulation activation. In addition, accumulating evidence indicates that obesity is a heterogeneous condition and not all individuals with obesity exhibit similar metabolic or thrombotic risk profiles. The concept of “metabolically healthy obesity” (MHO) challenges the assumption that all individuals with obesity are at equal cardiovascular risk [123]. Elucidating how distinct obesity phenotypes influence coagulation activation may therefore provide important insights into personalized risk stratification and targeted therapeutic strategies.

In conclusion, obesity is associated with a prothrombotic state characterized by increased coagulation activation and impaired fibrinolysis. This meta‐analysis provides robust evidence that key hemostatic parameters, including D‐dimer, fibrinogen, PAI‐1, vWF, FVIII, and ETP, are elevated in individuals with obesity, with particularly pronounced alterations observed in children. These findings highlight the potential importance of early intervention in obesity management, as addressing obesity at an early stage may help mitigate prothrombotic profiles related to obesity and may prevent long‐term thrombo‐embolic obesity‐related risk. Future studies should focus on elucidating the mechanistic pathways linking obesity and coagulation and on exploring targeted preventive and therapeutic strategies to address this growing public health challenge.

## Author Contributions

**Julia Buchold:** writing – review and editing, Data curation. Hans‐Jonas Meyer: writing – review and editing, methodology, data curation, conceptualization. **Manuel Florian Struck:** writing – review and editing. **Shrey Kohli:** writing – review and editing. **Akash Mathew:** review and editing. **Gyulten Mangova:** writing – review and editing. **Alexey Surov:** writing – review and editing, methodology. **Matthias Blüher:** writing – review and editing. **Berend Isermann:** writing – review and editing, writing – original draft, visualization. **Silke Zimmermann:** writing – review and editing, writing – original draft, visualization, methodology, formal analysis.

## Funding

B.I. was funded by the German Research Foundation (Deutsche Forschungsgemeinschaft, DFG) IS‐67/16‐1, IS‐67/22‐1, IS‐67/25‐1, IS‐67/26‐1; CRC1423 (project C07) and the ERC (101141561_TameThiflam); and the “Stiftung Pathobiochemie und Molekulare Diagnostik” (SPMD). S.Z. was funded by the Faculty of Medicine (Leipzig) (961000‐227). B.I. and M.B. were funded by the Deutsche Forschungsgemeinschaft (DFG, German Research Foundation) under Germany's Excellence Strategy (EXC‐3105/1–533765739).

## Conflicts of Interest

The authors declare no conflicts of interest.

## Supporting information


**Figure S1:** Forest plot of Std. Mean difference of age of children among studies. Due to nonavailability of respective SD in three studies, those could not be included here (Halle, Akinci, Rupérez).
**Figure S2:**. Funnel plot assessing publication bias for fibrinogen in adults for the included studies. The vertical line represents the pooled effect estimate, and dashed lines indicate the pseudo 95% confidence limits.
**Figure S3:**. Funnel plot assessing publication bias for fibrinogen in children for the included studies. The vertical line represents the pooled effect estimate, and dashed lines indicate the pseudo 95% confidence limits.
**Figure S4:**. Funnel plot assessing publication bias for PAI‐1 in children for the included studies. The vertical line represents the pooled effect estimate, and dashed lines indicate the pseudo 95% confidence limits.
**Figure S5:** Sensitivity analysis of D‐dimer: Forest plot of the meta‐analysis of standardized mean difference of D‐dimer by study group (obesity vs. control group). CI: confidence interval, df: degrees of freedom, SD: standard deviation
**Figure S6:** Sensitivity analysis of PAI‐1: Forest plot of the meta‐analysis of standardized mean difference of PAI‐1 by study group (obesity vs. control group). CI: confidence interval, df: degrees of freedom, SD: standard deviation
**Figure S7:** Sensitivity analysis of fibrinogen: Forest plot of the meta‐analysis of standardized mean difference of fibrinogen by study group (obesity vs. control group). CI: confidence interval, df: degrees of freedom, SD: standard deviation
**Figure S8:** Sensitivity analysis of fibrinogen after n.a. removal: Forest plot of the meta‐analysis of standardized mean difference of fibrinogen by study group (obesity vs. control group). CI: confidence interval, df: degrees of freedom, SD: standard deviation
**Table S1:**. Supporting Information.

## Data Availability

The original contributions presented in the study are included in the article. Further inquiries can be directed to the corresponding author.
