# Supplementary material for: Obesity‐Related Coagulation Activation in Adolescents and Children: A Systematic Review and Meta‐Analysis
Source: Obes Rev. 2026 Apr 26;27(10):e70137. doi: 10.1111/obr.70137 (PMC13551388; doi:10.1111/obr.70137)
Supplement: Supplementary file 1 [file OBR-27-e70137-s001.pdf]

Supplementary Information

Obesity-related coagulation activation in adolescents and children: a systematic review and meta-analysis

Julia Buchold[1#], Hans-Jonas Meyer[2#], Manuel Florian Struck[3], Shrey Kohli[1], Akash Mathew[1], Gyulten Mangova[1], Alexey Surov[4], Matthias Blüher[5,6,7,8], Berend Isermann[1,8#], Silke Zimmermann[1*#]

[1] Institute of Laboratory Medicine, Clinical Chemistry and Molecular Diagnostics, University of Leipzig, Leipzig, Germany
[2] Department of Diagnostic and Interventional Radiology, University of Leipzig, Leipzig, Germany
[3] Department of Anesthesiology and Intensive Care Medicine, University of Leipzig, Leipzig, Germany
[4] Department of Radiology, Neuroradiology and Nuclear Medicine, Johannes Wesling University Hospital, Ruhr University, Bochum, Germany
[5] Helmholtz Institute for Metabolic Obesity and Vascular Research (HI-MAG) of the Helmholtz Zentrum München at the University of Leipzig and University Hospital Leipzig, Leipzig, Germany
[6] Medical Department III - Endocrinology, Nephrology, and Rheumatology, Leipzig University Medical Center, Leipzig, Germany
[7] German Center for Child and Adolescent Health (DZKJ), Leipzig/Dresden partner site, Leipzig, Germany
[8] LeiCeM - Leipzig Center of Metabolism, Leipzig University, Leipzig, Germany

[#] Shared authorship
*Correspondence: silke.zimmermann@medizin.uni-leipzig.de
Dr. Dr. Silke Zimmermann
Paul-List-Str. 13/15
04103 Leipzig, Germany

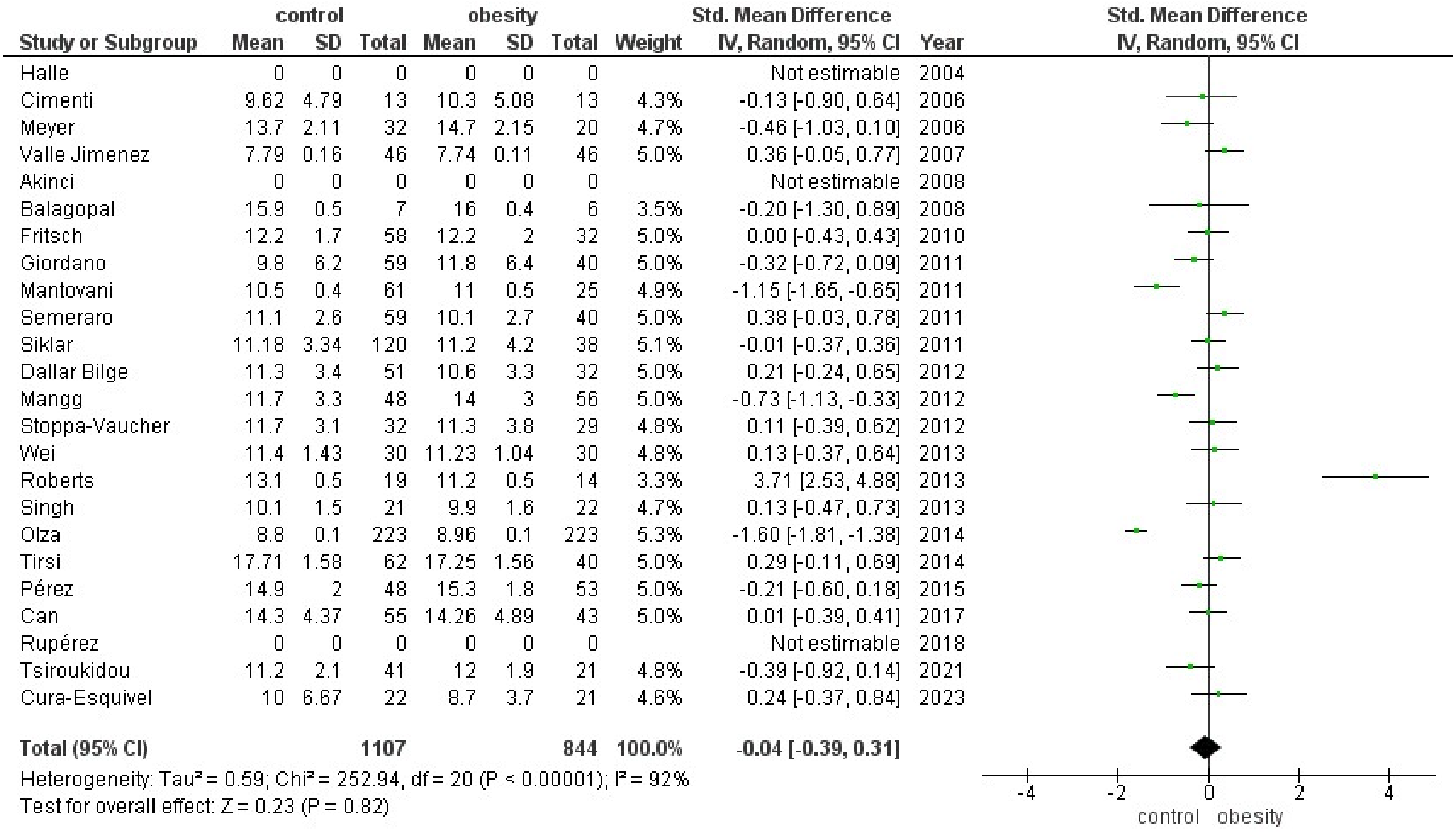

Study or Subgroup	control Mean	control SD	control Total	obesity Mean	obesity SD	obesity Total	Weight	Std. Mean Difference IV, Random, 95% CI	Year
Halle	0	0	0	0	0	0		Not estimable	2004
Cimenti	9.62	4.79	13	10.3	5.08	13	4.3%	-0.13 [-0.90, 0.64]	2006
Meyer	13.7	2.11	32	14.7	2.15	20	4.7%	-0.46 [-1.03, 0.10]	2006
Valle Jimenez	7.79	0.16	46	7.74	0.11	46	5.0%	0.36 [-0.05, 0.77]	2007
Akinci	0	0	0	0	0	0		Not estimable	2008
Balagopal	15.9	0.5	7	16	0.4	6	3.5%	-0.20 [-1.30, 0.89]	2008
Fritsch	12.2	1.7	58	12.2	2	32	5.0%	0.00 [-0.43, 0.43]	2010
Giordano	9.8	6.2	59	11.8	6.4	40	5.0%	-0.32 [-0.72, 0.09]	2011
Mantovani	10.5	0.4	61	11	0.5	25	4.9%	-1.15 [-1.65, -0.65]	2011
Semeraro	11.1	2.6	59	10.1	2.7	40	5.0%	0.38 [-0.03, 0.78]	2011
Siklar	11.18	3.34	120	11.2	4.2	38	5.1%	-0.01 [-0.37, 0.36]	2011
Dallar Bilge	11.3	3.4	51	10.6	3.3	32	5.0%	0.21 [-0.24, 0.65]	2012
Mangg	11.7	3.3	48	14	3	56	5.0%	-0.73 [-1.13, -0.33]	2012
Stoppa-Vaucher	11.7	3.1	32	11.3	3.8	29	4.8%	0.11 [-0.39, 0.62]	2012
Wei	11.4	1.43	30	11.23	1.04	30	4.8%	0.13 [-0.37, 0.64]	2013
Roberts	13.1	0.5	19	11.2	0.5	14	3.3%	3.71 [2.53, 4.88]	2013
Singh	10.1	1.5	21	9.9	1.6	22	4.7%	0.13 [-0.47, 0.73]	2013
Olza	8.8	0.1	223	8.96	0.1	223	5.3%	-1.60 [-1.81, -1.38]	2014
Tirsi	17.71	1.58	62	17.25	1.56	40	5.0%	0.29 [-0.11, 0.69]	2014
Pérez	14.9	2	48	15.3	1.8	53	5.0%	-0.21 [-0.60, 0.18]	2015
Can	14.3	4.37	55	14.26	4.89	43	5.0%	0.01 [-0.39, 0.41]	2017
Rupérez	0	0	0	0	0	0		Not estimable	2018
Tsiroukidou	11.2	2.1	41	12	1.9	21	4.8%	-0.39 [-0.92, 0.14]	2021
Cura-Esquivel	10	6.67	22	8.7	3.7	21	4.6%	0.24 [-0.37, 0.84]	2023
**Total (95% CI)**			**1107**			**844**	**100.0%**	**-0.04 [-0.39, 0.31]**	

Heterogeneity: Tau² = 0.59; Chi² = 252.94, df = 20 (P < 0.00001); I² = 92%
Test for overall effect: Z = 0.23 (P = 0.82)

Fig. S1. Forest plot of Std. Mean difference of age of children among studies. Due to non-availability of respective SD in three studies, those could not be included here (Halle, Akinci, Rupérez).

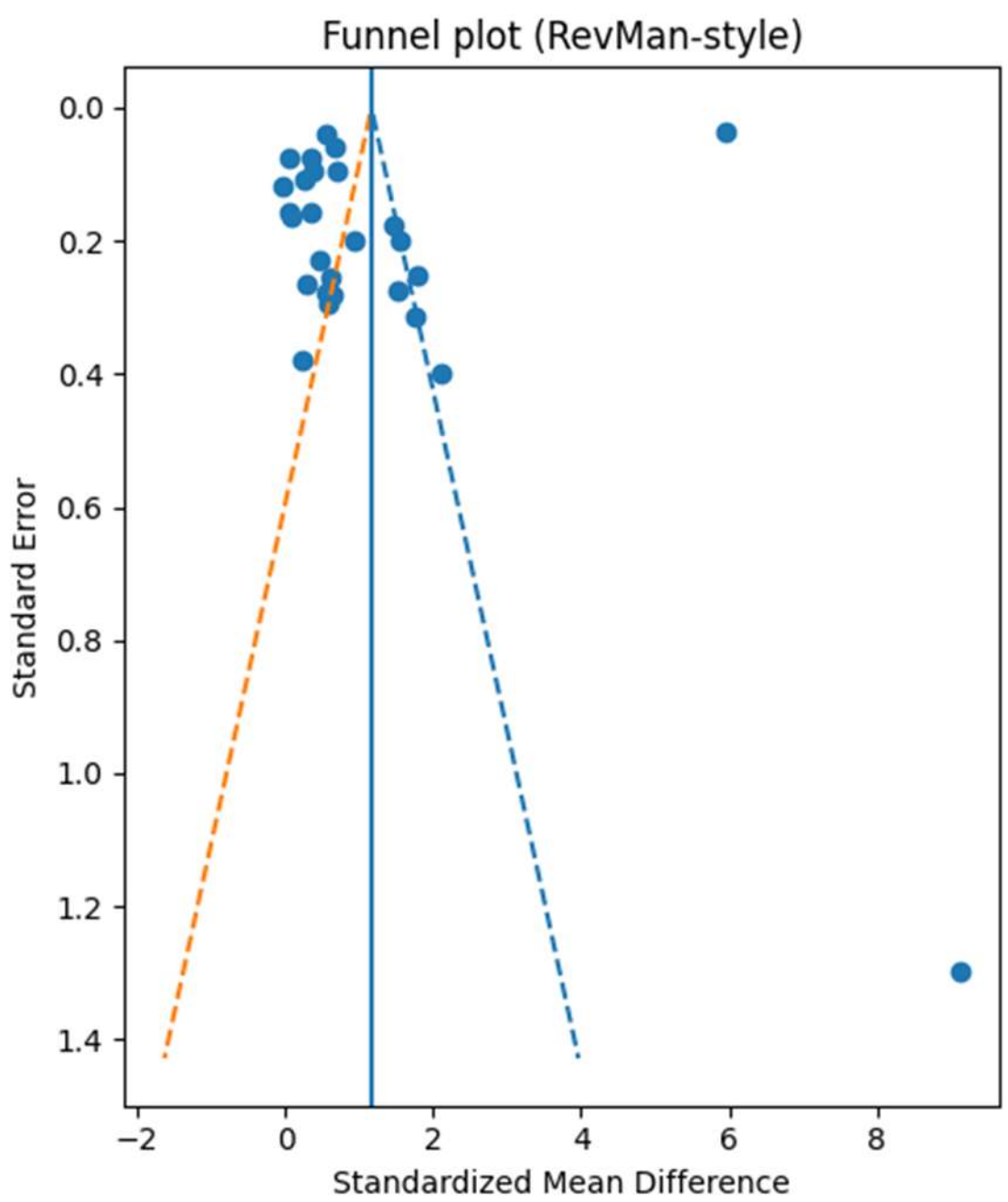

Fig. S2. Funnel plot assessing publication bias for fibrinogen in adults for the included studies. The vertical line represents the pooled effect estimate, and dashed lines indicate the pseudo 95% confidence limits.

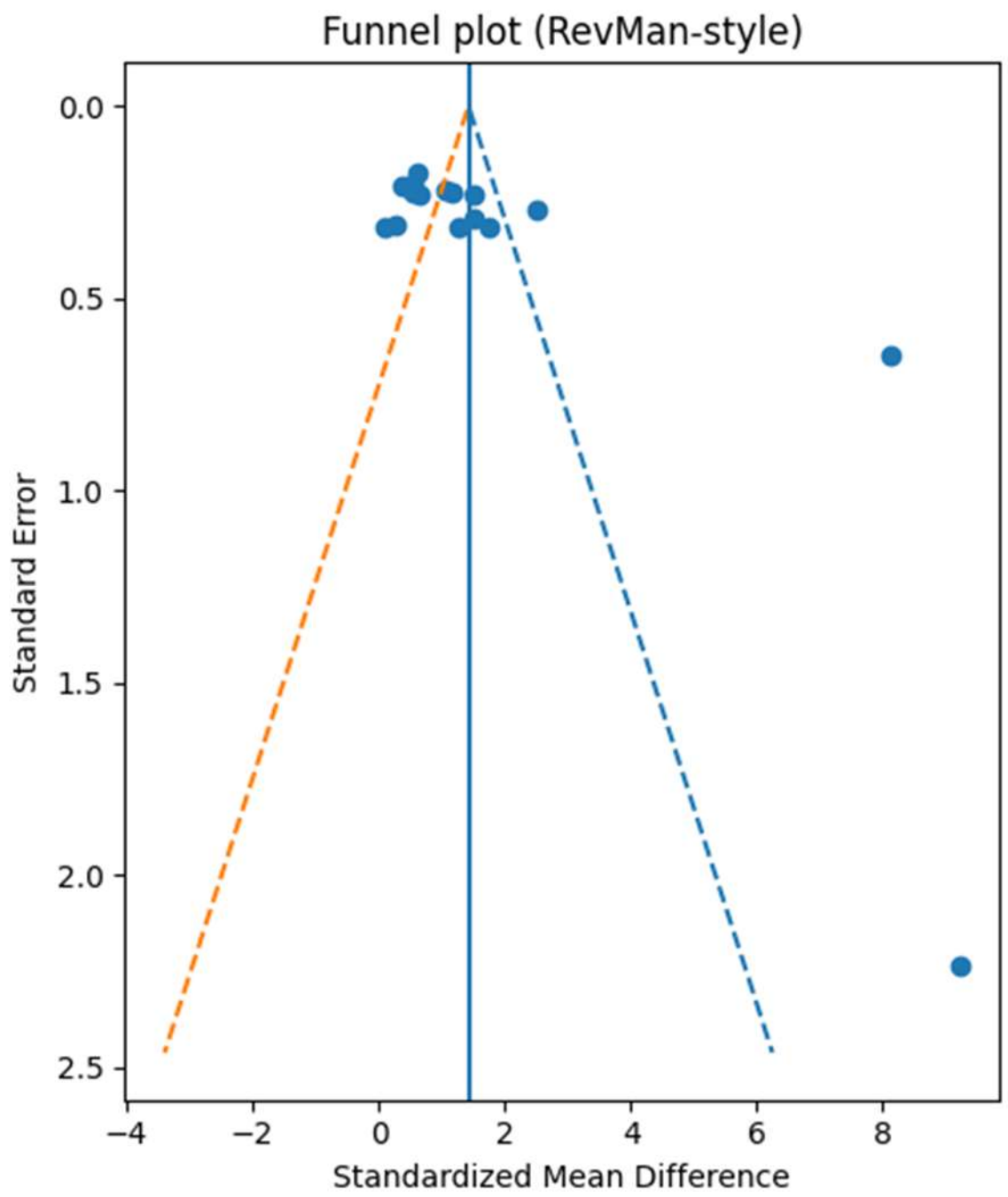

Fig. S3. Funnel plot assessing publication bias for fibrinogen in children for the included studies. The vertical line represents the pooled effect estimate, and dashed lines indicate the pseudo 95% confidence limits.

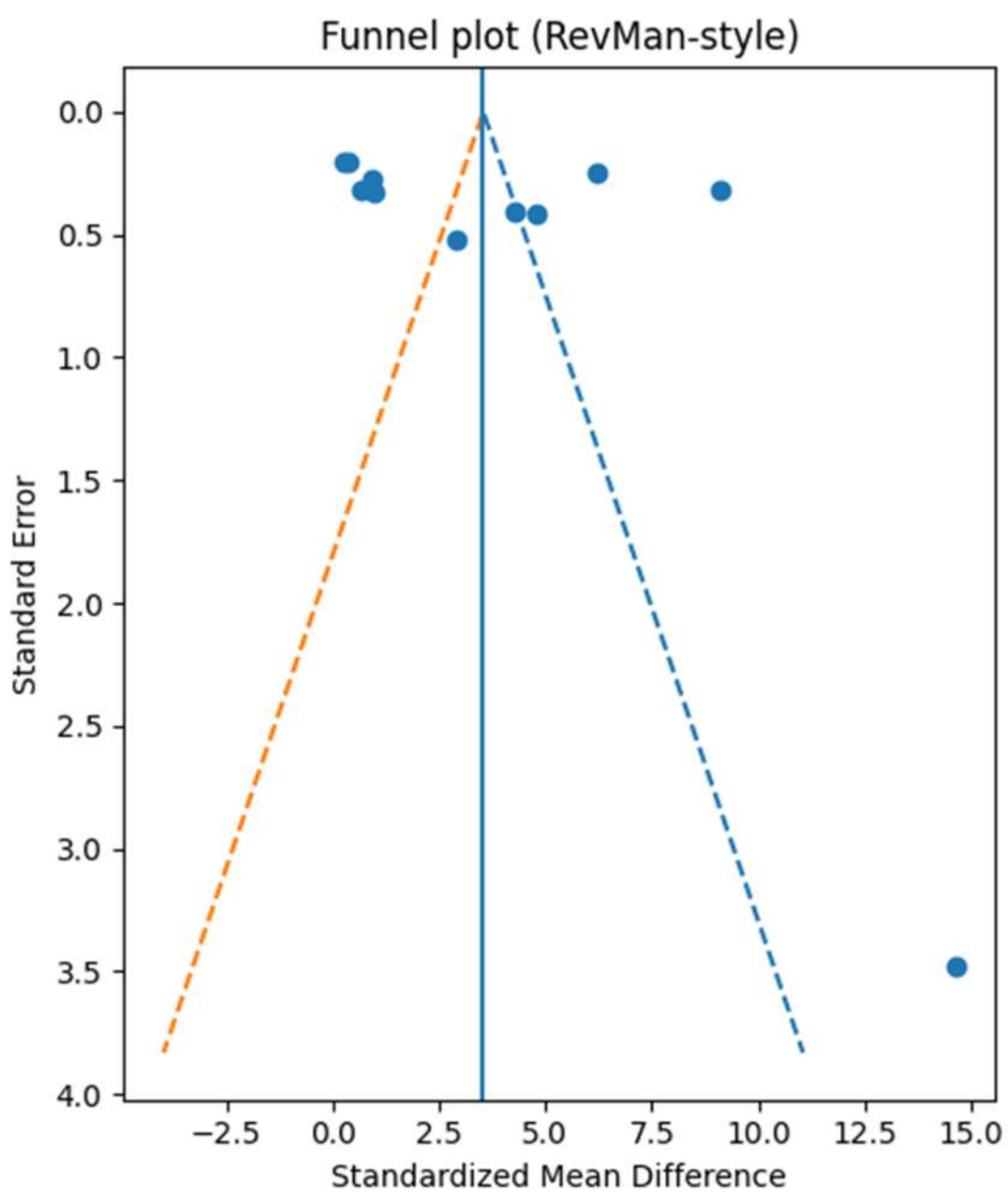

Fig. S4. Funnel plot assessing publication bias for PAI-1 in children for the included studies. The vertical line represents the pooled effect estimate, and dashed lines indicate the pseudo 95% confidence limits.

	experimental			control				Std. Mean Difference	
**Study or Subgroup**	**Mean**	**SD**	**Total**	**Mean**	**SD**	**Total**	**Weight**	**IV, Random, 95% CI**	**Year**
**1.1.1 adults**									
Taskiran	710.66	505.33	48	790.86	900.3	38	16.9%	-0.11 [-0.54, 0.31]	2010
Ceo	270	10	18	190	10	21	8.9%	7.84 [5.90, 9.78]	2012
Michalska	360	148	50	190	192	20	16.4%	1.04 [0.49, 1.59]	2013
Firszt-Adamczyk	182	85.93	36	95.44	95.7	24	16.4%	0.95 [0.40, 1.50]	2016
**Subtotal (95% CI)**			**152**			**103**	**58.6%**	**1.99 [0.50, 3.47]**	
Heterogeneity: Tau² = 2.06; Chi² = 68.18, df = 3 (P < 0.00001); I² = 96%									
Test for overall effect: Z = 2.62 (P = 0.009)									
**1.1.2 children**									
Balagopal	122.7	12.6	7	63.7	12.9	6	7.5%	4.31 [2.05, 6.57]	2008
Semeraro	549	362	59	345	230	40	16.9%	0.64 [0.23, 1.05]	2011
Giordano	403.3	579	59	249.9	159	40	17.0%	0.33 [-0.07, 0.74]	2011
**Subtotal (95% CI)**			**125**			**86**	**41.4%**	**0.93 [0.05, 1.80]**	
Heterogeneity: Tau² = 0.41; Chi² = 11.90, df = 2 (P = 0.003); I² = 83%									
Test for overall effect: Z = 2.07 (P = 0.04)									
**Total (95% CI)**			**277**			**189**	**100.0%**	**1.49 [0.67, 2.31]**	
Heterogeneity: Tau² = 0.99; Chi² = 80.35, df = 6 (P < 0.00001); I² = 93%									
Test for overall effect: Z = 3.57 (P = 0.0004)									
Test for subgroup differences: Chi² = 1.46, df = 1 (P = 0.23), I² = 31.3%									

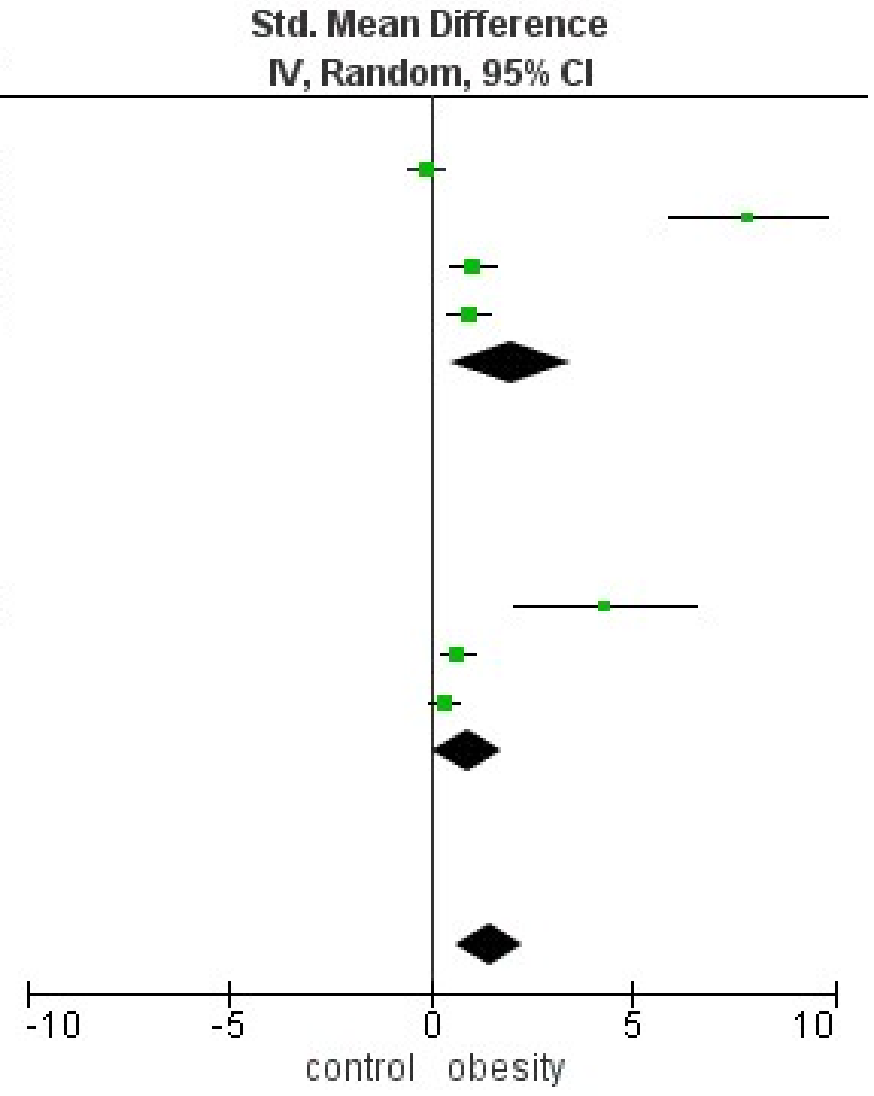

Fig. S5. Sensitivity analysis of D-dimer: Forest plot of the meta-analysis of standardized mean difference of D-dimer by study group (obesity vs. control group). CI: confidence interval, df: degrees of freedom, SD: standard deviation

Study or Subgroup	Experimental Mean	Experimental SD	Experimental Total	Control Mean	Control SD	Control Total	Weight	Std. Mean Difference IV, Random, 95% CI	Year
**1.3.1 adults**									
Taskiran	73.53	35.79	48	57.14	34.24	38	5.0%	0.46 [0.03, 0.89]	2010
Espino	10.63	4.82	50	14.26	11.4	71	5.1%	-0.39 [-0.75, -0.02]	2011
Pardina	160	15	34	75	10	22	4.5%	6.31 [4.99, 7.64]	2012
Ceo	60	6	18	20	2	21	3.8%	9.06 [6.85, 11.27]	2012
Wildman	24.8	59.3	330	17.2	44.5	384	5.1%	0.15 [-0.00, 0.29]	2012
Michalska	108	10.4	50	48	7.4	20	4.7%	6.14 [4.98, 7.31]	2013
Mhod Nor	57.82	49.22	182	43.74	30.65	98	5.1%	0.32 [0.07, 0.57]	2018
Lempesis	39.8	4.3	6	24.8	2.4	9	3.9%	4.32 [2.25, 6.40]	2023
**Subtotal (95% CI)**			**718**			**663**	**37.2%**	**2.68 [1.74, 3.62]**	
Heterogeneity: Tau² = 1.51; Chi² = 268.08, df = 7 (P < 0.00001); I² = 97%									
Test for overall effect: Z = 5.61 (P < 0.00001)									
**1.3.2 children**									
Valle Jimenez	30.95	2.39	46	20.51	1.9	46	4.9%	4.80 [3.98, 5.61]	2007
Balagopal	21.4	1.24	7	4.84	0.77	6	1.2%	14.63 [7.81, 21.46]	2008
Giordano	41.3	59.2	59	28.2	25	40	5.1%	0.27 [-0.13, 0.67]	2011
Semeraro	44.7	22.3	59	36.2	25.3	40	5.1%	0.36 [-0.05, 0.76]	2011
Mantovani	58.5	7.1	61	29.2	6	25	4.9%	4.27 [3.47, 5.07]	2011
Singh	37.3	18	21	25.6	15	22	5.0%	0.69 [0.08, 1.31]	2013
Wei	105.53	2.02	30	102.35	4.43	30	5.0%	0.91 [0.38, 1.45]	2013
Cura-Esquivel	27.61	10.5	22	19.18	5.4	21	5.0%	0.98 [0.35, 1.62]	2023
**Subtotal (95% CI)**			**305**			**230**	**36.0%**	**2.03 [0.88, 3.18]**	
Heterogeneity: Tau² = 2.37; Chi² = 184.90, df = 7 (P < 0.00001); I² = 96%									
Test for overall effect: Z = 3.46 (P = 0.0005)									
**1.3.3 gender-specific**									
Plaisance men	38.3	4.2	16	18.9	2.4	16	4.3%	5.53 [3.92, 7.13]	2020
Plaisance women	35.9	1.7	14	17.5	1.8	15	3.2%	10.20 [7.29, 13.12]	2020
**Subtotal (95% CI)**			**30**			**31**	**7.5%**	**7.70 [3.13, 12.27]**	
Heterogeneity: Tau² = 9.49; Chi² = 7.58, df = 1 (P = 0.006); I² = 87%									
Test for overall effect: Z = 3.30 (P = 0.0010)									
**1.3.4 gender-specific children**									
Mantovani girls	58.2	11.1	31	47.9	6.9	13	4.9%	1.00 [0.32, 1.69]	2011
Mantovani boys	58.9	9.1	30	9	6.1	12	4.4%	5.84 [4.37, 7.31]	2011
Olza boys	26.86	1.73	119	18.28	1.17	119	5.0%	5.79 [5.21, 6.37]	2014
Olza girls	30.71	2.01	104	18.89	1.21	104	4.9%	7.10 [6.36, 7.84]	2014
**Subtotal (95% CI)**			**284**			**248**	**19.3%**	**4.92 [2.03, 7.81]**	
Heterogeneity: Tau² = 8.46; Chi² = 168.31, df = 3 (P < 0.00001); I² = 98%									
Test for overall effect: Z = 3.34 (P = 0.0008)									
**Total (95% CI)**			**1337**			**1172**	**100.0%**	**3.40 [2.55, 4.25]**	
Heterogeneity: Tau² = 3.65; Chi² = 1213.87, df = 21 (P < 0.00001); I² = 98%									
Test for overall effect: Z = 7.87 (P < 0.00001)									
Test for subgroup differences: Chi² = 8.19, df = 3 (P = 0.04), I² = 63.4%									

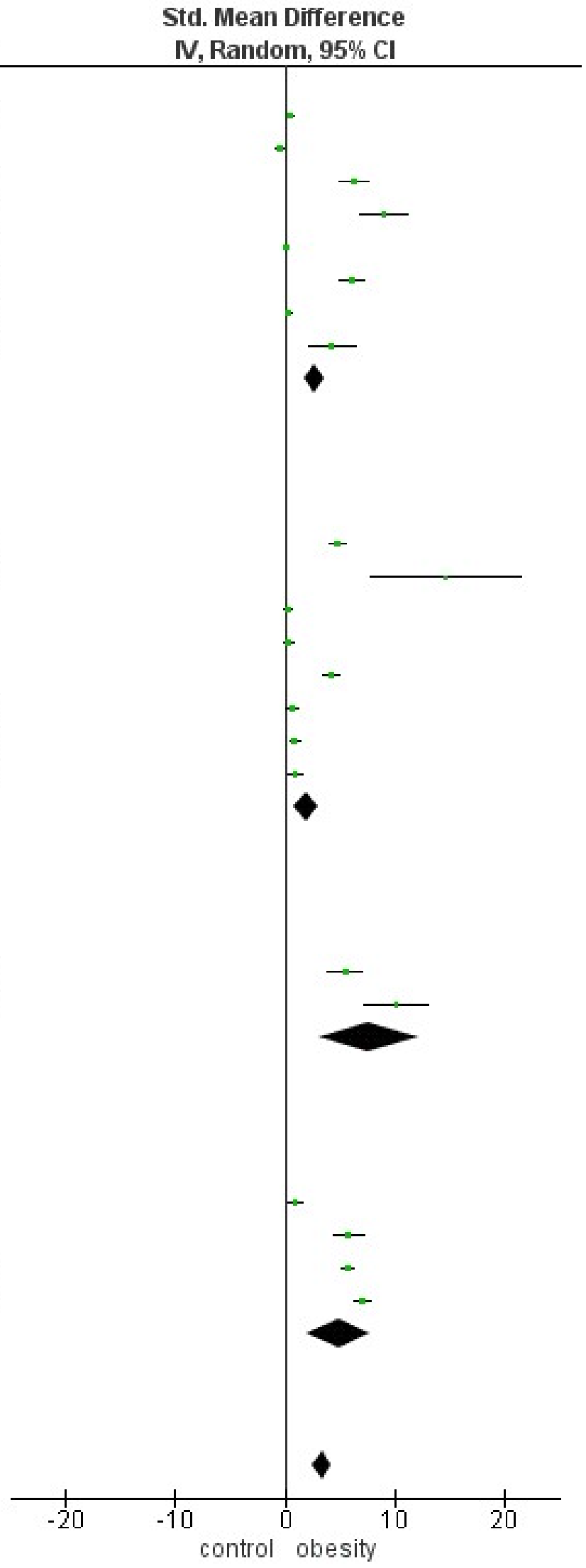

Fig. S6. Sensitivity analysis of PAI-1: Forest plot of the meta-analysis of standardized mean difference of PAI-1 by study group (obesity vs. control group). CI: confidence interval, df: degrees of freedom, SD: standard deviation

Study or Subgroup	Experimental Mean	Experimental SD	Experimental Total	Control Mean	Control SD	Control Total	Weight	Std. Mean Difference IV, Random, 95% CI	Year
**1.4.1 adults**									
Polac	4.36	1.06	20	2.6	0.48	21	2.1%	2.12 [1.34, 2.89]	2003
Gómez-Ambrosi	3.42	0.81	967	3.12	0.78	122	2.2%	0.37 [0.18, 0.56]	2006
Nguyen	3.77	0.03	3262	3.54	0.04	17571	2.2%	5.96 [5.89, 6.03]	2009
Calori	2.74	0.48	43	2.71	0.66	708	2.2%	0.05 [-0.26, 0.35]	2010
Wildman	2.79	2.22	330	2.67	1.78	384	2.2%	0.06 [-0.09, 0.21]	2012
Solá	3.21	0.51	67	2.51	0.37	67	2.2%	1.56 [1.17, 1.95]	2012
Kaye	3.4	0.89	14	3.2	0.81	14	2.1%	0.23 [-0.52, 0.97]	2012
Targher	3.22	0.5	45	2.41	0.4	45	2.2%	1.77 [1.28, 2.26]	2012
Cazettes	3.71	0.87	44	3.21	0.49	19	2.2%	0.64 [0.09, 1.19]	2012
Mangg	3.39	0.64	31	2.82	0.6	176	2.2%	0.94 [0.54, 1.33]	2012
Samocha-Bonet	3.33	0.77	30	2.94	0.48	35	2.2%	0.61 [0.11, 1.11]	2012
Moreira	3.44	0.79	21	2.98	0.78	28	2.1%	0.58 [-0.00, 1.16]	2013
Strohacker	3.43	0.82	440	3.16	0.76	298	2.2%	0.34 [0.19, 0.49]	2013
Hoekstra	3.11	1.2	96	3.16	2.39	268	2.2%	-0.02 [-0.26, 0.21]	2014
van Dongen	3	0.7	174	2.8	0.8	174	2.2%	0.27 [0.05, 0.48]	2015
de Oliviera	3.47	0.87	145	3.41	0.52	51	2.2%	0.08 [-0.24, 0.39]	2015
Campello	3.28	0.37	20	2.88	0.82	40	2.2%	0.56 [0.01, 1.11]	2015
Ilinčić	3.6	0.8	50	2.5	0.5	25	2.2%	1.52 [0.98, 2.06]	2016
Firszt-Adamczyk	3.2	0.92	36	2.96	0.71	24	2.2%	0.28 [-0.24, 0.80]	2016
Chitongo	4.2	0.8	89	3.1	0.7	77	2.2%	1.45 [1.11, 1.79]	2017
Mariani	3.84	0.74	75	3.5	0.7	26	2.2%	0.46 [0.01, 0.91]	2018
Meisel	3.3	0.8	1000	2.9	0.7	2366	2.2%	0.55 [0.47, 0.62]	2020
Rossi	3.15	0.67	61	2.93	0.63	123	2.2%	0.34 [0.03, 0.65]	2020
Tsiroukidou	3.79	0.78	42	2.45	0.68	21	2.1%	1.77 [1.15, 2.38]	2021
Bilooka	4.39	0.16	10	3.3	0.09	21	1.7%	9.13 [6.58, 11.67]	2021
Morcillo	3.177	0.58	235	2.786	0.51	229	2.2%	0.71 [0.53, 0.90]	2023
Bibi	3.2	0.74	602	2.7	0.74	620	2.2%	0.68 [0.56, 0.79]	2024
**Subtotal (95% CI)**			**7949**			**23553**	**58.0%**	**1.17 [0.14, 2.20]**	
Heterogeneity: $Tau^2 = 7.37$; $Chi^2 = 17674.76$, df = 26 ($P < 0.00001$); $I^2 = 100\%$									
Test for overall effect: Z = 2.22 (P = 0.03)									
**1.4.2 children**									
Halle	3.1	0.57	153	2.75	0.52	44	2.2%	0.62 [0.28, 0.96]	2004
Meyer	3.5	0.85	32	2.59	0.38	20	2.1%	1.26 [0.65, 1.88]	2006
Valle Jimenez	2.83	0.08	46	2.25	0.06	46	2.0%	8.13 [6.87, 9.40]	2007
Akinci	2.42	1.41	21	2.27	1.78	20	2.1%	0.09 [-0.52, 0.70]	2008
Balagopal	3.53	0.25	7	1.64	0.07	6	1.2%	9.23 [4.84, 13.61]	2008
Fritsch	3.3	0.74	58	2.95	0.44	32	2.2%	0.53 [0.09, 0.97]	2010
Giordano	2.43	1.88	59	1.76	1.68	40	2.2%	0.37 [-0.04, 0.77]	2011
Semeraro	2.44	0.5	59	1.86	0.48	40	2.2%	1.17 [0.74, 1.60]	2011
Mangg	3.34	0.89	48	3.01	0.1	56	2.2%	0.54 [0.15, 0.93]	2012
Dallar Bilge	3.64	0.69	51	3.2	0.63	32	2.2%	0.65 [0.20, 1.11]	2012
Stoppa-Vaucher	3.32	0.65	32	2.52	0.33	29	2.2%	1.51 [0.94, 2.08]	2012
Singh	3.21	0.45	21	3.07	0.6	22	2.1%	0.26 [-0.34, 0.86]	2013
Tirsi	3.61	0.9	62	2.82	0.39	40	2.2%	1.05 [0.63, 1.48]	2014
Pérez	3.94	0.7	48	2.45	0.47	53	2.2%	2.50 [1.98, 3.03]	2015
Can	3.73	0.88	55	2.63	0.47	43	2.2%	1.50 [1.04, 1.95]	2017
Tsiroukidou	3.789	0.784	42	2.45	0.678	21	2.1%	1.76 [1.15, 2.37]	2021
**Subtotal (95% CI)**			**794**			**544**	**33.4%**	**1.43 [0.93, 1.92]**	
Heterogeneity: $Tau^2 = 0.90$; $Chi^2 = 221.50$, df = 15 ($P < 0.00001$); $I^2 = 93\%$									
Test for overall effect: Z = 5.61 ($P < 0.00001$)									
**1.4.3 gender-specific**									
Tabassum women	3.39	0.69	584	2.91	0.72	584	2.2%	0.68 [0.56, 0.80]	2013
Tabassum men	3.13	0.78	488	2.86	0.71	488	2.2%	0.36 [0.24, 0.49]	2013
Plaisance women	2.95	0.22	14	2.69	0.23	15	2.1%	1.12 [0.33, 1.91]	2020
Plaisance men	2.69	0.17	16	2.9	0.28	16	2.1%	-0.88 [-1.61, -0.15]	2020
**Subtotal (95% CI)**			**1102**			**1103**	**8.6%**	**0.39 [0.02, 0.76]**	
Heterogeneity: $Tau^2 = 0.10$; $Chi^2 = 29.51$, df = 3 ($P < 0.00001$); $I^2 = 90\%$									
Test for overall effect: Z = 2.08 (P = 0.04)									
**Total (95% CI)**			**9845**			**25200**	**100.0%**	**1.27 [0.57, 1.98]**	
Heterogeneity: $Tau^2 = 5.91$; $Chi^2 = 19172.93$, df = 46 ($P < 0.00001$); $I^2 = 100\%$									
Test for overall effect: Z = 3.54 (P = 0.0004)									
Test for subgroup differences: $Chi^2 = 11.24$, df = 2 (P = 0.004), $I^2 = 82.2\%$									

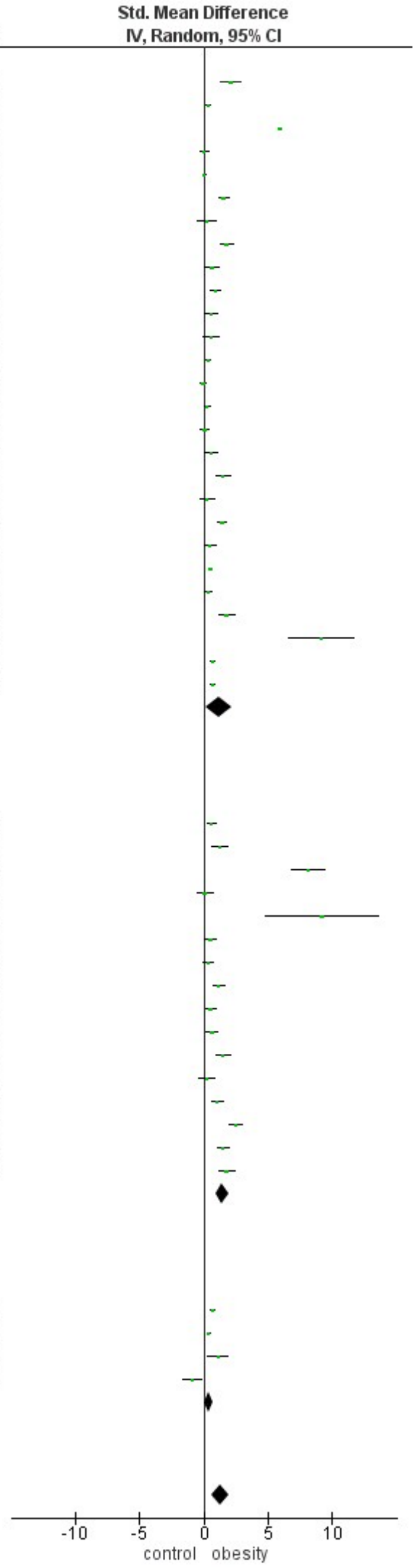

Fig. S7. Sensitivity analysis of fibrinogen: Forest plot of the meta-analysis of standardized mean difference of fibrinogen by study group (obesity vs. control group). CI: confidence interval, df: degrees of freedom, SD: standard deviation

Study or Subgroup	Experimental Mean	Experimental SD	Experimental Total	Control Mean	Control SD	Control Total	Weight	Std. Mean Difference IV, Random, 95% CI	Year
**1.4.1 adults**									
Polac	4.36	1.06	20	2.6	0.48	21	3.2%	2.12 [1.34, 2.89]	2003
Gómez-Ambrosi	3.42	0.81	967	3.12	0.78	122	3.2%	0.37 [0.18, 0.56]	2006
Nguyen	3.77	0.03	3262	3.54	0.04	17571	3.3%	5.96 [5.89, 6.03]	2009
Wildman	2.79	2.22	330	2.67	1.78	384	3.2%	0.06 [-0.09, 0.21]	2012
Solá	3.21	0.51	67	2.51	0.37	67	3.2%	1.56 [1.17, 1.95]	2012
Kaye	3.4	0.89	14	3.2	0.81	14	3.2%	0.23 [-0.52, 0.97]	2012
Cazettes	3.71	0.87	44	3.21	0.49	19	3.2%	0.64 [0.09, 1.19]	2012
Mangg	3.39	0.64	31	2.82	0.6	176	3.2%	0.94 [0.54, 1.33]	2012
Samocha-Bonet	3.33	0.77	30	2.94	0.48	35	3.2%	0.61 [0.11, 1.11]	2012
Moreira	3.44	0.79	21	2.98	0.78	28	3.2%	0.58 [-0.00, 1.16]	2013
van Dongen	3	0.7	174	2.8	0.8	174	3.2%	0.27 [0.05, 0.48]	2015
de Oliviera	3.47	0.87	145	3.41	0.52	51	3.2%	0.08 [-0.24, 0.39]	2015
Campello	3.28	0.37	20	2.88	0.82	40	3.2%	0.56 [0.01, 1.11]	2015
Ilinčić	3.6	0.8	50	2.5	0.5	25	3.2%	1.52 [0.98, 2.06]	2016
Firszt-Adamczyk	3.2	0.92	36	2.96	0.71	24	3.2%	0.28 [-0.24, 0.80]	2016
Chitongo	4.2	0.8	89	3.1	0.7	77	3.2%	1.45 [1.11, 1.79]	2017
Tsiroukidou	3.79	0.78	42	2.45	0.68	21	3.2%	1.77 [1.15, 2.38]	2021
Morcillo	3.177	0.58	235	2.786	0.51	229	3.2%	0.71 [0.53, 0.90]	2023
Bibi	3.2	0.74	602	2.7	0.74	620	3.2%	0.68 [0.56, 0.79]	2024
**Subtotal (95% CI)**			**6179**			**19698**	**61.4%**	**1.07 [-0.34, 2.48]**	
Heterogeneity: Tau² = 9.78; Chi² = 13318.57, df = 18 (P < 0.00001); I² = 100%									
Test for overall effect: Z = 1.49 (P = 0.14)									
**1.4.2 children**									
Valle Jimenez	2.83	0.08	46	2.25	0.06	46	3.1%	8.13 [6.87, 9.40]	2007
Akinci	2.42	1.41	21	2.27	1.78	20	3.2%	0.09 [-0.52, 0.70]	2008
Fritsch	3.3	0.74	58	2.95	0.44	32	3.2%	0.53 [0.09, 0.97]	2010
Giordano	2.43	1.88	59	1.76	1.68	40	3.2%	0.37 [-0.04, 0.77]	2011
Mangg	3.34	0.89	48	3.01	0.1	56	3.2%	0.54 [0.15, 0.93]	2012
Dallar Bilge	3.64	0.69	51	3.2	0.63	32	3.2%	0.65 [0.20, 1.11]	2012
Stoppa-Vaucher	3.32	0.65	32	2.52	0.33	29	3.2%	1.51 [0.94, 2.08]	2012
Singh	3.21	0.45	21	3.07	0.6	22	3.2%	0.26 [-0.34, 0.86]	2013
Tirsi	3.61	0.9	62	2.82	0.39	40	3.2%	1.05 [0.63, 1.48]	2014
Pérez	3.94	0.7	48	2.45	0.47	53	3.2%	2.50 [1.98, 3.03]	2015
Can	3.73	0.88	55	2.63	0.47	43	3.2%	1.50 [1.04, 1.95]	2017
Tsiroukidou	3.789	0.784	42	2.45	0.678	21	3.2%	1.76 [1.15, 2.37]	2021
**Subtotal (95% CI)**			**543**			**434**	**38.6%**	**1.45 [0.82, 2.07]**	
Heterogeneity: Tau² = 1.14; Chi² = 201.97, df = 11 (P < 0.00001); I² = 95%									
Test for overall effect: Z = 4.53 (P < 0.00001)									
**1.4.3 gender-specific**									
**Subtotal (95% CI)**			**0**			**0**		**Not estimable**	
Heterogeneity: Not applicable									
Test for overall effect: Not applicable									
**Total (95% CI)**			**6722**			**20132**	**100.0%**	**1.26 [0.20, 2.31]**	
Heterogeneity: Tau² = 8.92; Chi² = 14186.35, df = 30 (P < 0.00001); I² = 100%									
Test for overall effect: Z = 2.34 (P = 0.02)									
Test for subgroup differences: Chi² = 0.23, df = 1 (P = 0.63), I² = 0%									

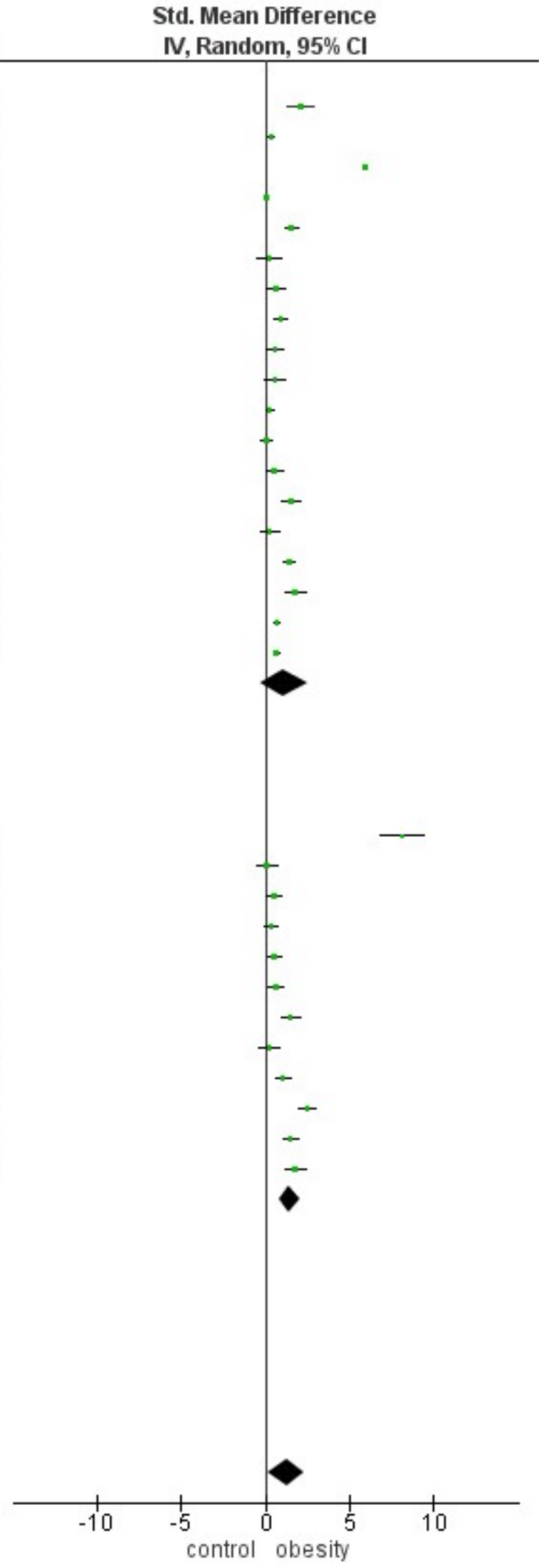

Fig. S8. Sensitivity analysis of fibrinogen after n.a. removal: Forest plot of the meta-analysis of standardized mean difference of fibrinogen by study group (obesity vs. control group). CI: confidence interval, df: degrees of freedom, SD: standard deviation

Table S1

Parameter	First Author	Year	Assay
D-Dimer	Michalska	2013	ELISA
D-Dimer	Morcillo	2023	Particle-enhanced immunoturbidimetric assay (PETIA)
D-Dimer	Semeraro	2011	ELISA
D-Dimer	Balagopal	2008	ELISA
D-Dimer	Ceo	2012	ELISA
D-Dimer	Firszt-Adamczyk	2016	ELISA
D-Dimer	Giordano	2011	ELISA
D-Dimer	Stoppa-Vaucher	2012	ELFA (Enzyme-Linked Fluorescent Assay)
D-Dimer	Taskiran	2010	ELISA
Fibrinogen	Balagopal	2008	Nephelometry
Fibrinogen	Bibi	2024	Claus
Fibrinogen	Bilooka	2021	n.a.
Fibrinogen	Campello	2014	Claus
Fibrinogen	Can	2017	Claus/derived
Fibrinogen	Chitongo	2017	Claus
Fibrinogen	Dallar Bilge	2012	Claus/derived
Fibrinogen	Ilinčić	2016	Claus/derived
Fibrinogen	Mariani	2018	n.a.
Fibrinogen	Nguyen	2009	Claus
Fibrinogen	Oliveira	2015	Claus/derived
Fibrinogen	Rossi	2020	n.a.
Fibrinogen	Samocha-Bonet	2012	Claus
Fibrinogen	Stoppa-Vaucher	2012	Claus/derived
Fibrinogen	Tabassum	2013	n.a.
Fibrinogen	van Dongen	2015	Claus
Fibrinogen	Akinci	2008	Claus
Fibrinogen	Calori	2010	n.a.
Fibrinogen	Cazettes	2012	Claus
Fibrinogen	Firszt-Adamczyk	2016	Claus
Fibrinogen	Fritsch	2010	Claus
Fibrinogen	Giordano	2011	Claus
Fibrinogen	Gómez-Ambrosi	2006	Claus
Fibrinogen	Halle	2004	n.a.
Fibrinogen	Hoekstra	2014	n.a.
Fibrinogen	Kaye	2012	Claus/derived
Fibrinogen	Mangg	2012	Claus
Fibrinogen	Meisel	2020	n.a.
Fibrinogen	Meyer	2006	n.a.
Fibrinogen	Morcillo	2023	Claus
Fibrinogen	Moreira	2013	Claus
Fibrinogen	Murakami	2007	n.a.
Fibrinogen	Pérez	2015	Immunoassay
Fibrinogen	Plaisance	2020	n.a.
Fibrinogen	Polac	2003	Claus
Fibrinogen	Semeraro	2011	n.a.

Fibrinogen	Singh	2013	Claus/derived
Fibrinogen	Solá	2012	Claus/derived
Fibrinogen	Strohacker	2013	Nephelometry
Fibrinogen	Targher	2012	Nephelometry
Fibrinogen	Tirsi	2014	Claus
Fibrinogen	Tsiroukidou	2021	Claus
Fibrinogen	Valle Jimenez	2007	Claus/derived
Fibrinogen	Wildman	2012	Claus/derived
PAI-1	Lempesis	2023	ELISA
PAI-1	Michalska	2013	ELISA
PAI-1	Mohd Nor	2018	ELISA
PAI-1	Rupérez	2018	n.a.
PAI-1	Semeraro	2011	ELISA
PAI-1	Singh	2013	ELISA
PAI-1	Wildman	2012	ELISA
PAI-1	Balagopal	2008	ELISA
PAI-1	Ceo	2012	ELISA
PAI-1	Cura–Esquivel	2023	ELISA
PAI-1	Espino	2011	ELISA
PAI-1	Mantovani	2011	ELISA
PAI-1	de Oliveira	2015	Bead-based multiplex sandwich immunoassay
PAI-1	Olza	2014	Particle-enhanced turbidimetric immunoassay
PAI-1	Pardina	2012	ELISA
PAI-1	Plaisance	2020	n.a.
PAI-1	Roberts	2013	bead-based multiplex sandwich immunoassay
PAI-1	Wei	2013	ELISA
PAI-1	Taskiran	2010	ELISA
PAI-1	Giordano	2011	ELISA
PAI-1	Olza	2012	Bead-based multiplex sandwich immunoassay
PAI-1	Valle Jimenez	2007	Immunoassay
vWF	Villalpando Sánchez	2022	ELISA
vWF	Patel	2016	ELISA
vWF	Singh	2013	ELISA
FVIII	Kaye	2012	Clotting
FVIII	Campello	2014	Clotting
FVIII	Dallar Bilge	2012	Clotting
FVIII	Morcillo	2023	Clotting
ETP	Campello	2014	CAT (Calibrated Automated Thrombogram)
ETP	Chitongo	2017	CAT
ETP	Fritsch	2010	CAT
ETP	Calderara	2020	CAT
ETP	de Laat-Kremers	2022	CAT
ETP	Cimenti	2006	CAT
ETP	Siklar	2011	CAT
